# Solid-state NMR spectroscopy reveals unique properties of *Trichoderma harzianum* cell wall components

**DOI:** 10.1016/j.tcsw.2025.100156

**Published:** 2025-10-11

**Authors:** A.A. Safeer, F.E.L. Kleijburg, H.A.B. Wösten, M. Baldus

**Affiliations:** a NMR Spectroscopy, Bijvoet Centre for Biomolecular Research, Utrecht University, Padualaan 8, 3584 CH Utrecht, the Netherlands; b Microbiology, Department of Biology, Utrecht University, Padualaan 8, 3584 CH Utrecht, the Netherlands

**Keywords:** NMR, *Trichoderma harzianum*, Mycoparasite, Fungal cell wall, Polysaccharide

## Abstract

*Trichoderma harzianum* is a saprophyte and a mycoparasite and is also capable of forming symbiotic connections with plants. This fungus interacts with the (a)biotic environment through its cell wall and as a mycoparasite secretes enzymes that degrade the cell wall polymers of its target fungi. The organization of the *T*. *harzianum* cell wall is not well known. We used solid-state NMR and Fourier transform infrared spectroscopy to probe the molecular composition and architecture of the *T*. *harzianum* cell wall at the atomic level. Our results revealed that the inner core of the *T. harzianum* rigid cell wall phase is largely composed of chitin, which is complemented with a more mobile cell wall layer that contains β-(1,3)-glucan. The outer dynamic phase of the cell wall is mainly composed of α- and β-glucans, arabinan, mannan and proteins. The relative abundance of both rigid and dynamic cell wall components changed when *T*. *harzianum* was grown on isolated fungal cell wall material instead of glucose. Our results suggest that *T. harzianum* forms a cell wall that is chemically distinct from other fungal species to prevent harmful self-digestion by its secreted lytic enzymes that do degrade the cell wall of target fungi.

## Nomenclature

**Glossary**
CP: Cross polarization
FTIR: Fourier transform infrared spectroscopy
DIPSI: Decoupling in the presence of scalar interactions
INEPT: Insensitive nuclei enhanced by polarization transfer
MAS: Magic Angle Spinning
MISSIPPI: Multiple Intense Solvent Suppression Intended for Sensitive Spectroscopic Investigation of Protonated Proteins, Instantly
HSQC: Heteronuclear Single Quantum Coherence
ssNMR: solid-state NMR
SPINAL64: small phase incremental alternation, with 64 steps
SPC5: Supercycled permutationally offset stabilized variant of C5
PISSARO: phase-inverted supercycled sequence for attenuation of rotary resonance
TOBSY: Total through-bond correlation spectroscopy
WALTZ 16: Wideband alternating-phase low-power technique for zero residual splitting 16

## 1 Introduction

The ascomycete *Trichoderma harzianum* is used as a biological control agent against phytopathogenic fungi and for plant growth promotion and biofortification (Tyskiewicz et al., 2022). The fungus can grow as a saprophyte, establishes mutual beneficial symbiotic interactions with plants, and acts as a mycoparasite (Harman et al., 2004). Mycoparasitism is initiated with the detection of the host, which involves the release of cell wall chitin oligomers from the target fungus by a secreted exochitinase from *T. harzianum* (Harman et al., 2004). The released oligomers induce the production of endochitinases that attack the cell wall of the target fungus. Next, *T. harzianum* attaches to the target fungus. To this end, carbohydrates in the *T. harzianum* cell wall bind to lectins in the cell wall of the host. *T. harzianum* hyphae coil around the fungus and form appressoria that mediate penetration of the host (Mukherjee et al., 2022). Cell wall degrading enzymes that are released by the mycoparasite contribute to the penetration (Mukherjee et al., 2022). This penetration combined with the release of antifungal compounds results in the killing of the hyphae of the host.

The basidiomycete *Schizophyllum commune* (Kleijburg and Wösten, 2025) is a target of *T. harzianum. S. commune* can only be co-cultured for a limited amount of time with *T. harzianum* (Wu et al., 2021) because its cell wall is degraded by the enzymes that are released by the mycoparasite (de Vries, 1974; de Vries and Wessels, 1973). Clearly, the cell wall degrading enzymes should not lyse the cell wall of *T. harzianum* itself. It was proposed that, in particular, chitin should be shielded to protect the cell wall of *T. harzianum* against self-degradation (Gruber and Seidl-Seiboth, 2012). This shielding could be achieved by chitin binding proteins. One of such proteins may be QID74 (Rosado et al., 2007). Protection of the cell wall of *T. harzianum* against self-degradation may also be achieved by differences in its molecular composition compared to target fungi such as *S. commune.*

So far, research on the *T*. *harzianum* cell wall composition employed destructive methods to study isolated polysaccharides outside of their native arrangement by solution-state nuclear magnetic resonance (NMR) spectroscopy (Saravanakumar et al., 2021). Such studies require extraction methods including ultrasonication, boiling, and precipitation and therefore modify the structural and dynamical architecture of the wall. These data thus lack information on the structural organization of the intact *T*. *harzianum* cell wall. Solid-state nuclear magnetic resonance (ssNMR) spectroscopy offers increasing possibilities to study the molecular composition and architecture of fungal cell walls in their native conformation at the atomic level (Camacho et al., 2019; Chatterjee et al., 2015; Chrissian et al., 2020; Ehren et al., 2020; Ghassemi et al., 2022; Kang et al., 2018; Zhong et al., 2008) and their interaction with molecules in the environment (Bishoyi et al., 2025; Fernando et al., 2023; Kleijburg et al., 2023; Safeer et al., 2023). For example, recent carbon-detected ssNMR studies on the human pathogens *Aspergillus fumigatus,* an ascomycete, and *Rhizopus delemar,* a zygomycete, were vital in establishing the architecture, structure, and dynamic changes of their cell wall (Chakraborty et al., 2021; Cheng et al., 2024; Kang et al., 2018; Lamon et al., 2023). In parallel, our groups have previously used ssNMR to study the composition of the cell wall of the basidiomycete *S. commune* (Ehren et al., 2020; Safeer et al., 2023) and its interaction with metal ions and antifungal peptides (Bishoyi et al., 2025; Kleijburg et al., 2023; Safeer et al., 2023). We developed proton-detected ssNMR experiments (Bahri et al., 2023; Safeer et al., 2023) that allowed us to resolve changes in the cell wall in an additional spectral dimension without the need of isotope labelling (Pylkkänen et al., 2023) and to dissect the relative polysaccharide abundance. Our studies revealed that the cell wall of *S. commune* contains a rigid inner core of α-(1,3)-glucan, β-(1,3)-(1,6)-glucan, highly branched and single stranded β-(1,4)-chitin, polymeric fucose and mannose. The α-(1,3)-glucan was found to be the most abundant polysaccharide in this part of the cell wall. The outer mobile cell wall fraction mostly consists of short α- and β-glucan chains together with longer β-(1,3)(1,6)-glucan (Ehren et al., 2020; Safeer et al., 2023). Solid-state NMR also revealed that the mobile *S. commune* cell wall domain contains proteins, and flexible and rigid lipid acyl chain signals that likely originate from the plasma membrane (Ehren et al., 2020; Safeer et al., 2023).

In the current study, we combined ^1^H and ^13^C-detected ssNMR correlation experiments with ^1^H-detected relaxation (Lewandowski et al., 2011) and water-edited ^13^C-detected (Ader et al., 2008; Weingarth et al., 2012) ssNMR approaches as well as Fourier transform infrared spectroscopy (FTIR) to reveal the composition, dynamics and architecture of the *T. harzianum* T22 cell wall*. T. harzianum* strain T22 is the main component in various biopesticides and biofertilizers (van Hee et al., 2025). The rigid cell wall consists of a chitin inner core and a more mobile β-(1,3)-glucan layer, while a flexible polysaccharide layer forms the outer part of the cell wall. T22 cultivation on isolated *S. commune* cell walls, instead of glucose as carbon source, reduced rigid polysaccharide abundance. Our results show that the cell wall composition of *T. harzianum* is distinct from that of *S. commune* as well as from *A. fumigatus,* which could play a critical role in protection against self-degradation of the mycoparasite.

## 2 Materials and methods

### 2.1 Solid-state NMR spectroscopy

NMR spectroscopy exploits the magnetic properties of non-zero nuclear spins and their behavior in an external magnetic field. Radio-frequency (r.f.) irradiation can be used to retrieve spectroscopic signals of these spins and to manipulate their interactions with the chemical environment and other nearby spins. Solid-state NMR (ssNMR) in combination with magic-angle spinning (MAS) (Andrew et al., 1958), allowed us to obtain high-resolution ^13^C and ^1^H NMR spectra of the cell wall sample without the need of extraction and solubilization methods that would be required for solution-state NMR. In the context of our current studies, we used this ssNMR setup to derive quantitative and qualitative information about the molecular composition of the cell wall by determining NMR resonance frequencies (given in ppm) as well as by establishing spin-spin proximities to reveal the chemical structure of the different molecular species as well as their dynamic behavior in two spectral dimensions. For the latter studies we utilized dedicated r.f. pulse-scheme experiments that establish through-space (dipolar) and through-bond (scalar) spin-spin interactions that have been shown to reveal rigid (exhibiting motion slower than on millisecond time scale) and dynamic (motion in the ns to low μs regime) molecules (see e.g. (Andronesi et al., 2005; Baldus and Meier, 1996)), respectively. Lastly, we analyzed local dynamic properties as well as the water-exposure of rigid cell wall materials using dedicated ssNMR schemes. To reduce spectral ambiguities, we acquired both ^13^C-detected and ^1^H-detected NMR experiments of ^13^C,^15^N-labeled *T*. *harzianum* T22 cell walls on a narrow-bore Avance III 700 MHz (16.4 T, Bruker Biospin, Billerica, MA, USA) spectrometer.

### 2.2 Culture conditions

*T*. *harzianum* strain T22 (ATCC 20847) was grown on a polycarbonate track etched membrane (PC; 0.1 μm pore size, diameter 76 mm) (GVS, Bologna, Italy) on top of an ammonium minimal medium (MM-N) (Kleijburg et al., 2023) 1.5 % agar plate. MM-N consisted of 20 g·L^−1^ glucose, 1.31 g·L^−1^ (NH_4_)_2_SO_4_, 0.5 g·L^−1^ MgSO_4_.7H_2_O, 1 g K_2_HPO_4_, 0.46 g KH_2_PO_4_, 0.12 mg·L^−1^ thiamine-HCl, 5 mg·L^−1^ FeCl_3_.6H_2_O, as well as trace elements. To achieve stable isotope labelling, glucose and (NH_4_)_2_SO_4_ were replaced by ^13^C_6_ glucose (Cortecnet, France) and (^15^NH_4_)_2_SO_4_ (Sigma-Aldrich, St Louis, MO, USA). Alternatively, T22 was grown on a PC membrane overlaying 60 mg water-washed U—^13^C,^15^N (ssNMR) or unlabeled (FTIR) cell walls from *S. commune* that were saturated with MQ (Safeer et al., 2023). Plates were inoculated with an agar plug from the periphery of a 7-day old colony and grown in a sealed plastic bag (20 × 30 cm) in the dark at 30 °C for 4 days.

*S. commune* strain H4-8 A (matA43 matB41; FGSC no. 9210) (Ohm et al., 2010) was grown for 7 days in the dark at 30 °C on a PC membrane overlaying a 1.5 % agar MM-N plate (Kleijburg et al., 2023). Cultures were inoculated with an agar plug from the periphery of a 7-day old colony and grown in a sealed plastic bag (20 × 30 cm). Liquid shaken cultures were inoculated by macerating a quarter of a 7-day-old colony in 50 mL MM-N for 30 s at 18·10^3^ rpm using a Waring 2 Speed Blender (Waring, Stamford, CT, USA). After incubation for 24 h at 30 °C and 200 rpm in a 250 mL Erlenmeyer, cultures were macerated again for 30 s. Next, 50 mL MM-N cultures were inoculated with 0.1 g wet weight mycelium homogenate and grown for 7 days at 30 °C and 200 rpm in 250 mL Erlenmeyers. For stable isotope labelling of liquid and static *S. commune* cultures, glucose and (NH_4_)_2_SO_4_ were replaced by ^13^C_6_ glucose (Cortecnet, France) and (^15^NH_4_)_2_SO_4_ (Sigma-Aldrich, St Louis, MO, USA).

### 2.3 Cell wall isolation for NMR spectroscopy and FTIR

Mycelium of *T*. *harzianum* T22 was harvested from the PC membrane with tweezers, freeze dried, and homogenized in a 15 mL Falcon tube with five 4.76 mm (85 mg) metal beads (Bofix, Wijk bij Duurstede, The Netherlands) for 9 min in an SK550 1.1 heavy-duty paint shaker (Fast & Fluid, Sassenheim, The Netherlands). The homogenate was washed four times with 10 mL dH_2_O with an intermittent 10-min centrifugation step at 1·10^4^ *g* resulting in water-washed cell walls.

Water-washed cell walls of *S. commune* were prepared in a similar way as water-washed cell walls from *T*. *harzianum* T22. In the case of liquid shaken cultures mycelium was isolated from 50 mL MM-N cultures by centrifugation (13.7·10^3^ *g* for 5 min). Half of the water-washed cell walls of *S. commune* were incubated twice for 20 min in 10 mL 1 M KOH and washed three times with 10 mL dH_2_O with intermittent centrifugation (see above) resulting in KOH-extracted cell walls.

### 2.4 Solid-state NMR spectroscopy

For ssNMR, water-washed cell walls of *T. harzianum T22* were resuspended in MQ and loaded into 1.3 mm and 3.2 mm magic angle spinning (MAS) rotors for ^1^H-detected and ^13^C-detected experiments, respectively. The caps of 1.3 mm rotors were sealed with nail polish to prevent sample dehydration during fast MAS. MISSISSIPPI (Zhou and Rienstra, 2008) water-suppression was applied for a duration of 120 ms and 200 ms at 23 kHz r.f. field strength during dipolar-based and scalar-based two-dimensional ^1^H—^13^C correlation experiments, respectively, using a 1.3 mm HXY MAS probe (Bruker BioSpin). Recycle delays for scalar- and dipolar based ^1^H-detected experiments were set to 1.4 s and 1.0 s, respectively. For all scalar-based HSQC (Bodenhausen and Ruben, 1980; Morris and Freeman, 1979) experiments, WALTZ16 (Shaka et al., 1983) decoupling was applied at 10 kHz on ^1^H and ^13^C channels and the ^13^C offset was set at 51 ppm with a spectral width of 130 ppm. For scalar-based hC(*c*)H experiments an additional DIPSI (Paluch et al., 2022; Shaka et al., 1988) C—C mixing at 17.5 kHz r.f. was applied for a 5.4 ms mixing time (Bahri et al., 2023). For all dipolar-based hCH (Paulson et al., 2003) experiments, ramped (70–100 %) forward and backward cross-polarization (CP) steps from ^1^H to ^13^C were applied with respective contact times of 1.2 ms and 0.2 ms, to ensure that only correlations would arise from directly bonded proton‑carbon pairs. In addition, PISSARRO (Weingarth et al., 2008) decoupling was applied on ^1^H and ^13^C channels at 15 kHz and the ^13^C offset was set at 57.3 ppm with a spectral width of 130 ppm for all dipolar-based experiments. The ^13^C-T_1ρ_ relaxation experiments (Lewandowski et al., 2011) for *T*. *harzianum* T22 and *S. commune* H4-8 A cell wall samples were recorded as dipolar-based 2D ^1^H—^13^C correlation spectra (see above) with a 17.5 kHz spin-lock on the ^13^C channel during delays of 0, 1, 2, 4, 8, 16, 24 and 32 ms. The sample was spun at an MAS rate of 54 kHz with a set temperature of 258 K.

^13^C-detected ssNMR experiments of *T*. *harzianum* T22 and *S. commune* cell walls were acquired using a 3.2 mm HCN Efree MAS probe (Bruker BioSpin). For all ^13^C-detected experiments, a recycle delay of 2 s was used and SPINAL64 (Fung et al., 2000) decoupling was used at 85 kHz on the ^1^H channel during acquisition. For the identification of rigid cell wall species, dipolar based double-quantum single-quantum (DQSQ) (Hohwy et al., 1999) 2D ^13^C—^13^C experiments were recorded of isolated *S. commune* and *T*. *harzianum* T22 cell wall material. SPC5 recoupling (Hohwy et al., 1999) was used at 40 kHz for a total of 0.4 ms mixing time with simultaneous continuous wave decoupling at 85 kHz. SPINAL64 (Fung et al., 2000) decoupling was used at 85 kHz on the ^1^H channel during acquisition. The ^13^C offset was 86 ppm with 304 ppm and 280 ppm spectral widths in the direct and indirect dimensions. For the identification of mobile cell wall species, a 2D ^13^C—^13^C INEPT-TOBSY (Andronesi et al., 2005; Baldus and Meier, 1996) experiment was recorded of *T*. *harzianum* T22 cell walls using 6.0 ms mixing time. Additionally, the ^13^C offset was set at 60 ppm with 250 ppm and 200 ppm spectral widths in the direct and indirect dimensions. Water-accessibility of the rigid components was determined by recording a series of water-edited (Ader et al., 2008; Weingarth et al., 2012) 1D CP experiments at 15 kHz MAS and a set temperature of 275 K. A *T*_2_-filter of 0.5 ms was applied and a mixing time range was used of 0–64 ms. Here, the ^13^C offset was 95 ppm with a 394 ppm spectral width and 9.9 ms of direct acquisition time (^13^C). For the 2D ^13^C-detected water-edited surface topology experiment (Ader et al., 2008; Weingarth et al., 2012), a 0.5 ms *T*_2_-filter was applied with a mixing time of 5 ms. The ^13^C and ^1^H offsets were 95 ppm and 2.6 ppm with respective spectral widths of 394 ppm and 12 ppm.

TopSpin version 4.1.0 (Bruker BioSpin) was used to process acquired data. Additional ^1^H- and ^13^C-detection acquisition parameters per sample, e.g., pulse power, MAS and set temperature Additional processing parameters are summarized per *T*. *harzianum* sample in **Table S1**. For all ^1^H-detected, INEPT-TOBSY, and water-edited experiments, actual sample temperatures were roughly room temperature due to the frictional heating during MAS, as calibrated using a KBr powder sample (Thurber and Tycko, 2009).

### 2.5 Solid-state NMR experimental data analysis

For the analysis of NMR spectra, chemical shift assignments of polysaccharide species were made using the Complex Carbohydrate Magnetic Resonance Database (Kang et al., 2020). For dynamics and water-edited experiments all curve fitting and half-time determination was performed using GraphPad Prism 9.5.1. Squared F-tests were used to determine two-phase decay significance of the relaxation decay fits (at *p < 0.05*). Errors of the input normalized intensities from one-dimensional water build-up experiments and errors of the normalized integrals from two-dimensional T_1ρ_ experiments were determined on the basis of the experimental NMR signal-to-noise using TopSpin 4.1 (Bruker Biospin). Error of the resulting water-edited build-up times and relaxation times were based on the output values of the fit. For relative abundances, error was also based on experimental signal-to-noise of the two-dimensional experiment and standard error-propagation techniques were applied. A more detailed description of the ssNMR data processing and relative abundance analysis, as well as the the acquisition parameters, fit parameters and output values are provided in the Supporting Information.

### 2.6 FITR *experimental data analysis*

For FTIR, a VerTex 70 Fourier-Transform Infrared (FTIR) Spectrometer (Bruker) was used to determine spectra of freeze dried water-washed and KOH-extracted cell walls between 4000 and 550 cm^−1^ at 1 cm^−1^ intervals. Data were analyzed using two-sample *t*-tests (in case of equal variance) or Welch two-sample t-tests (in case of unequal variance) (*p* ≤ 0.05). Biological triplicates were used and each sample was measured three times.

## 3 Results

### 3.1 The rigid cell wall phase consists of separate inflexible chitin and mobile β-(1,3)-glucan layers

**Fig. 1:**
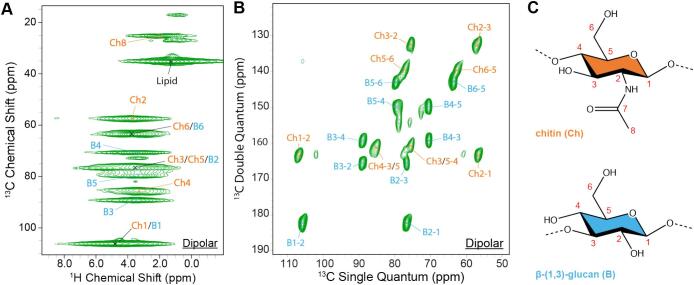
ssNMR-guided identification of *T*. *harzianum* T22 rigid cell wall components. **A)** Dipolar based ^1^H—^13^C correlation 2D spectrum of water-washed cell walls. **B)**^13^C-detected Double-Quantum Single-Quantum solid-state NMR spectra for the assignment of the rigid cell wall components. **C)** Molecular structures of the monomers of identified rigid polysaccharides.

^1^H-detected ssNMR spectroscopy was employed for the characterization of water-washed *T*. *harzianum* T22 cell walls. The cell wall material was isolated from uniformly ^13^C,^15^N-labeled mycelium. We recorded a dipolar-based ^1^H—^13^C correlation experiment (Fig. 1A) that employs through space dipolar couplings between nearby nuclear spins to probe the rigid cell wall components. The assignments were based on dipolar ^13^C—^13^C correlation spectra Fig. 1B, **Tables S2** - **S3**) and ^13^C chemical shift values from literature (Ehren et al., 2020; Kang et al., 2020). Remarkably, we found that only β-(1,3)-glucan (B) and chitin (Ch; β-(1,4)-*N*-acetylglucosamine) form the rigid cell wall phase of *T. harzianum* (see Fig. 1C for structures). Notably, previous ssNMR experiments revealed dominating α-glucan contributions for both *S. commune* (Ehren et al., 2020) and *A. fumigatus* (Bishoyi et al., 2025; Gautam et al., 2025; Kang et al., 2018)*.* A strong bulk lipid signal was also observed in the rigid cell wall fraction, which likely originates from the acyl chains in the co-isolated plasma membrane. Similar to our observations for the *S. commune* cell wall (Safeer et al., 2023), the large ^1^H linewidth for all resonances is likely the result of a combination of inhomogeneous and homogenous line broadening, and ultra-high field studies are required to further reduce spectral overlap for the C1 and C6 resonances in the ^1^H dimension (Fig. 1A ). In contrast to *A. fumigatus* (Bishoyi et al., 2025; Chakraborty et al., 2021) rigid amino acid signals were not detected, which indicates that proteins are of minor structural importance for the rigid cell wall organization of *T*. *harzianum*.

Previous studies have suggested that the presence of chitosan, the deacetylated form of chitin, as well as the degree of deacetylation (Kasaai, 2010) of chitin play a principal role in maintaining cell wall plasticity in various fungal species (Baker et al., 2007; Latge, 2007; Lesage and Bussey, 2006). Interestingly and unlike in other ssNMR studies (see, e.g. (Bishoyi et al., 2025)), isolated chitosan resonances seem to be completely absent in both [^1^H,^13^C] as well as [^13^C,^13^C] correlation spectra of *T*. *harzianum* T22 cell walls (Fig. 1A and 1B). While spectral overlap of chitin and β-(1,3)-glucan backbone peaks precludes determination of the degree of deacetylation according to the established ssNMR method (Heux et al., 2000; Kasaai, 2010; Manni et al., 2010; Ottøy et al., 1996; Pelletier et al., 1990), comparison of the integrated peaks between the backbone Ch2 or Ch4 resonances with acetyl Ch8 resonance peak (**Table S4**) revealed little difference. Taken together, these findings suggest a near full acetylation of cell wall chitin in *T*. *harzianum* T22*.* Interestingly, for other mycoparasitic *Trichoderma* species such as *T. viride* and *T*. *atroviride*, the presence of chitosan and/or deacetylated chitin has been reported along with an important role for chitosan in mycoparasitism (Hu et al., 2004; Kappel et al., 2024).

**Fig. 2:**
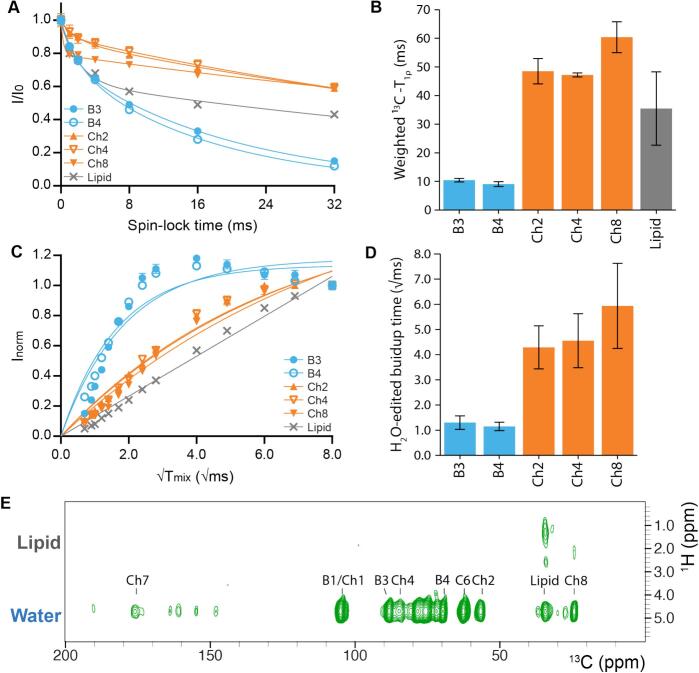
Dynamics and water-accessibility of rigid cell wall components of *T*. *harzianum* T22. **A)** Integrals of peaks from dipolar 2D ^1^H—^13^C correlation spectra from ^13^C-T_1ρ_ relaxation experiments were normalized and fitted with a bi-exponential. **B)** Weighted ^13^C-T_1ρ_ relaxation times for rigid cell wall components under a ^13^C spin-lock in dipolar-based 2D ^1^H—^13^C correlation spectra. Error is based on fit. **C)** Signal intensities from ^13^C-detected 1D water-edited CP experiments were normalized and fitted with a mono-exponential. **D)** Signal buildup times from water-edited ^13^C-detected 1D spectra demonstrate the water-accessibility for the rigid cell wall components of *T*. *harzianum* T22. **E)** Topology of the rigid cell wall probed by dipolar-based water-edited ^13^C—^1^H 2D correlation spectrum using a mixing time of 5 ms (see Materials and Methods section as well as the supporting information for details including determination of errors bars). Abbreviations in (A) and (B) indicate chitin (Ch; orange) and β-(1,3)-glucan (B; blue). (For interpretation of the references to colour in this figure legend, the reader is referred to the web version of this article.)

Next, we recorded a series of two-dimensional ^1^H-detected experiments with an increasing ^13^C spin-lock time (Figs. 2A and **S1**) to study the dynamics of the rigid components of water-washed cell walls of *T*. *harzianum* T22 (Chill et al., 2006; Lewandowski, 2013; Lewandowski et al., 2011). The resulting weighted ^13^C T_1ρ_ times for the sampled resonances are shown in Fig. 2B. Slow signal decay for chitin resonances (orange, Fig. 2B) gives rise to the high T_1ρ_ values and indicates low mobility of chitin. Meanwhile, β-(1,3)-glucan signals (blue, Fig. 2B) decay quickly, as denoted by low T_1ρ_, which indicates a much higher degree of mobility in the cell wall. A high degree of β-(1,3)-glucan mobility was previously also reported for *A. fumigatus* (Kang et al., 2018). As the T_1ρ_ series signal decays are fit best by a biexponential (Fig. 2A), β-(1,3)-glucan and chitin seem to possess both fast and slow decay populations (see per resonance population in **Table S5**). This observation suggests the existence of at least two different species for both β-(1,3)-glucan and chitin that exhibit different dynamical properties. In fact, the heterogeneity in dynamical behavior could originate from an interface in the rigid cell wall phase between chitin and β-glucan layers where a population of chitin and β-(1,3)-glucan chains may intersperse and collectively exhibit faster or slower dynamics. This would agree with the view that (at least part of the) β-(1,3)-glucan and chitin form a cohesive, mesh-like structure (Latge, 2007).

Next, we performed a series of one-dimensional water-edited ^13^C-detected ssNMR cross-polarization experiments (Figs. 2C and **S2**) (Ader et al., 2008; Weingarth et al., 2012) to investigate the water-accessibility of the *T*. *harzianum* rigid cell wall components in hydrated material (see Materials and Methods). These experiments provide insight into the relative distance of rigid cell wall components from the surface water. The water-edited signal buildup times for the sampled mixing times are shown per resonance in Fig. 2D. Here, curves were fitted with mono-exponentials (**Table S6**). We observe a slow buildup of rigid chitin signal, which suggests low accessibility to water molecules (orange, Fig. 2D). On the other hand, rigid β-(1,3)-glucan signals seem to build up fast indicating higher accessibility to water and thus likely a shorter distance to the surface than for chitin. Taken together, these observations emphasize that– unlike the similar build-up times for *S. commune* cell wall components indicate (**Fig. S3**) – major portions of β-(1,3)-glucan and chitin are layered separately in the T22 rigid cell wall phase and suggest that the β-(1,3)-glucan stratum is possibly more porous than the chitin core. Notably, we also observe that the probed rigid lipid signal is still near linearly increasing at a mixing time of 64 ms (8 √ms, Fig. 2D), indicating that lipids are located distant from the hydrated surface.

Using a two-dimensional water-edited ^13^C—^1^H topology experiment (Ader et al., 2008; Weingarth et al., 2012), we were able to track ^13^C species that are in contact with the H_2_O or lipid tail resonances. We did not observe cross-peaks between the polysaccharide and lipid tail resonances (Fig. 2E). The absence of detectable polysaccharide to lipid tail contacts could be caused by shielding of efficient signal transfer due to the continued presence of an intact plasma membrane lipid bilayer or the emergence of sufficiently large micelles in our homogenized water-washed cell wall sample.

### 3.2 A diverse layer of polysaccharides forms the flexible cell wall phase

**Fig. 3:**
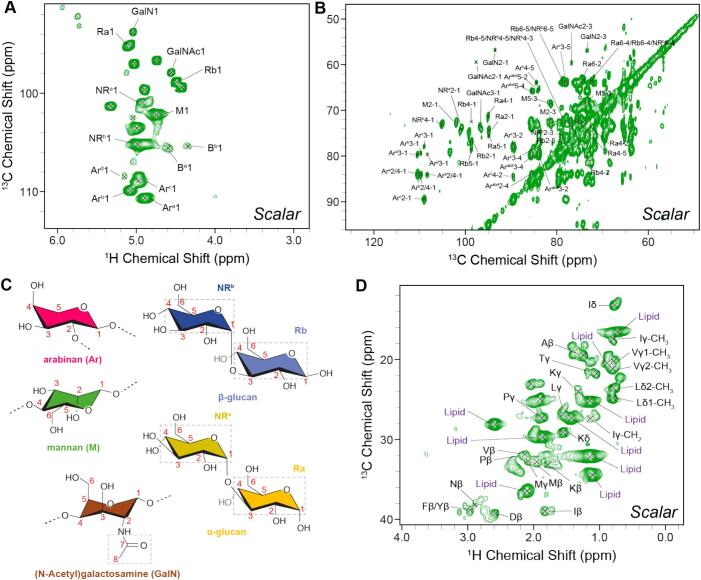
Characterization of the flexible cell wall components of *T*. *harzianum* T22. **A)** The polysaccharide signals observed in a scalar-based ^1^H—^13^C correlation 2D spectrum of water-washed cell walls. **B)** Spectral cutout of the polysaccharide bulk region from a 2D ^13^C—^13^C INEPT-TOBSY (Andronesi et al., 2005; Baldus and Meier, 1996) spectrum recorded with 5 ms of mixing time. **C)** Molecular structures of the monomers of identified flexible polysaccharides. **D)** Spectral cutout of the scalar-based ^1^H—^13^C correlation experiment, revealing the presence of dynamic amino acids/proteins and lipid correlations*.* Correlations of amino acids are given in single letter notation. See **Fig. S5** for full scalar-based spectra.

We next conducted scalar based ^13^C- and ^1^H-detected 2D ssNMR experiments that use *J*-couplings between bonded nuclear spins to assign flexible *T*. *harzianum* T22 cell wall components (Fig. 3A, B and **S4**). Assignments (**Tables S7** - **S8**) were made with assistance of the Complex Carbohydrates Magnetic Resonance Database and literature values for *S. commune*, for which a high degree of similarity was found (Ehren et al., 2020; Kang et al., 2020; Safeer et al., 2023). Notably, we observed that a branched arabinose containing polysaccharide (Kang et al., 2018; Lee et al., 2005) is a major contributor to the flexible cell wall which is in line with previous studies (Saravanakumar et al., 2021) that conducted an HPLC analysis on *T. harzianum*. Notably, arabinose was not found by NMR in the targeted model system *S. commune* (Ehren et al., 2020). Based on the high chemical shift value of the arabinan C2, this polysaccharide likely contains 1,2-linkages (Kang et al., 2018; Perez Garcia et al., 2011; Wang et al., 2014). Furthermore, the flexible cell wall polysaccharides consist of β-glucans and α-glucans. Both types of polysaccharides include reducing end species that are likely short and flexible as described for *S. commune* (see Fig. 3C for structures) (Ehren et al., 2020; Safeer et al., 2023). As with *S. commune*, we observed dynamic amino acids signals (Fig. 3D) stemming from hydrophobic (I, L, V, A, P, F), polar (Y, T) but also charged amino acids (K) that likely originate from non-covalently bound proteins and could even belong to cysteine-rich hydrophobins, even though no NMR resonances indicative of cysteine resonance were observed.

### 3.3 Growth on fungal cell wall material diminishes polysaccharide abundance

**Fig. 4:**
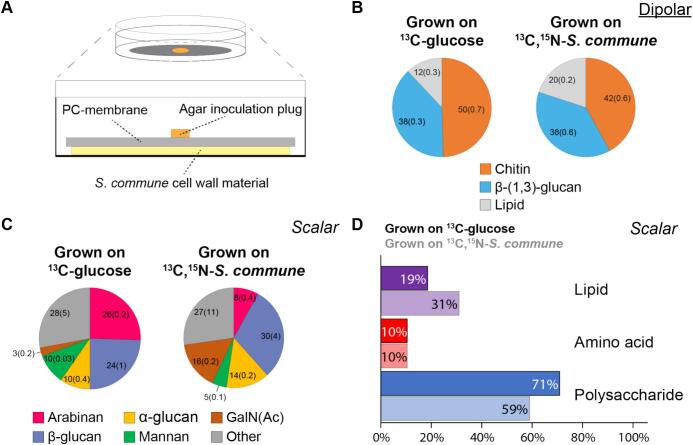
The effect of growth medium on the rigid *T*. *harzianum* T22 cell wall composition. **A)** Set-up of the growth of *T*. *harzianum* T22 on [U-^13^C,^15^N]-labeled *S. commune* cell walls. **B)** Relative abundance of *T*. *harzianum* T22 rigid cell wall components based on ^1^H-detected 2D spectra of isolated cell wall material. Errors based on signal-to-noise of the integrals are shown in brackets. **C)** Relative abundance of flexible polysaccharide species. Errors based on signal-to-noise are shown in brackets. **D)** Contribution per component type to all scalar cell wall signals for *T*. *harzianum* T22 based on 2D ^1^H—^13^C correlation spectrum. Top bars reflect contributions under growth on glucose while bottom bars reflect contributions under growth on *S. commune* cell walls.

To study the impact of changing the nutrient source on the composition of the *T*. *harzianum* cell wall, *T*. *harzianum* T22 was grown on hydrated water-washed U—^13^C,^15^N-labeled cell wall material isolated from *S. commune*. The water-washed cell walls from *T*. *harzianum* T22 mycelium produced in the experimental set-up shown schematically in Fig. 4A, was studied using ^1^H-detected ssNMR (**Fig. S5, S6**). The relative abundance of the rigid cell wall components (Fig. 4B **and Fig. S6**, **Table S9**) for both growth methods was determined by integrating the peaks resulting from the dipolar-based ^1^H—^13^C correlation experiments (Safeer et al., 2023). We observed limited compositional difference when comparing the rigid cell wall of *T*. *harzianum* T22 grown on MM-N with glucose and on *S. commune* cell walls. We find that in both growth conditions the amount of rigid β-(1,3)-glucan is 38 % (Fig. 4B), while the rigid chitin content decreases from 50 % to 42 % upon growth of T22 on *S. commune* cell walls (Fig. 4B). Interestingly, the cell wall of *T*. *harzianum* grown on *S. commune* cell walls has a higher rigid lipid contribution (20 % versus 12 %). As the bulk lipid signal likely originates from the plasma membrane and is thus unlikely to change in abundance, we speculate that the total rigid polysaccharides abundance decreases and a thinner rigid cell wall for *T*. *harzianum* T22 is formed when grown on fungal cell wall material. The decrease in rigid chitin could be the result of adaptation to the available growth substrate or an indication that rigid chitin is partially digested by the secreted lytic enzymes (de Vries and Wessels, 1973), likely during biosynthesis (Vermeulen and Wessels, 1986). Notably, the chitin content for *T*. *harzianum* T22 is considerably higher than for *S. commune* and *A. fumigatus* (Bishoyi et al., 2025; Chakraborty et al., 2021; Safeer et al., 2023), which is line with earlier findings of high chitin content in the cell wall of mycoparasitic species in the *Trichoderma* genus (Kappel and Gruber, 2020). Increasing the chitin content in the cell wall could be a method for T22 to circumvent harmful side-effects of self-digestion of chitin by its secreted lytic enzyme mixture.

Direct comparison shows that cell wall material from *T*. *harzianum* T22 lacks the rigid α-(1,3)-glucan core present in the *S. commune* (Ehren et al., 2020) and *A. fumigatus* (Gautam et al., 2025; Kang et al., 2018) cell walls. *Fusarium oxysporum* is a common target of *T*. *harzianum* in soil when used in agricultural application (Innocenti et al., 2015) and also contains α-(1,3)-glucan as a rigid, condensed alkali-insoluble polysaccharide (Schoffelmeer et al., 1999). It is likely that the absence of rigid α-(1,3)-glucan is crucial to the mycoparasitic function of *T*. *harzianum* as this polysaccharide is a primary target for cell wall digestion by secreted α-glucanases (de Vries and Wessels, 1973).

Similar to the rigid cell wall phase, the type of scalar (flexible) polysaccharide components in the cell walls of *T*. *harzianum* T22 was mostly similar between growth on glucose and water-washed *S. commune* cell walls (**Fig. S5**). The relative abundance of the flexible polysaccharides in the water-washed cell walls of *T*. *harzianum* T22 was determined by analyzing the cumulative C1 peak heights (**Table S10**). Notably, we observed a significant decrease in flexible arabinan abundance following growth on water-washed *S. commune* cell walls (26 % to 8 %, Fig. 4C). By analyzing the cumulative peak heights of all resonances in a scalar 2D ^1^H—^13^C spectrum the majority of the flexible *T*. *harzianum* T22 cell wall was shown to consist of polysaccharides at 71 % (Fig. 4D), which is substantially lower than the 91 % reported for static *S. commune* cell wall material (Kleijburg, 2025). The flexible lipid acyl tail signal made up 19 % of signals followed by amino acid signals (Fig. 4D), likely part of polypeptides, at 10 %. However, growth of *T*. *harzianum* on *S. commune* cell wall material (Fig. 4D) leads to an increase in flexible lipid signal abundance from 19 % to 31 % and a decrease in flexible polysaccharide abundance from 71 % to 59 %. This effect could indicate self-digestion of flexible polysaccharides and thus a thinner flexible polysaccharide layer, similar to what is observed for the rigid cell wall phase.

It has been proposed that *T*. *harzianum* prevents cell wall self-digestion by coating cell wall polysaccharides with protein (Rosado et al., 2007). The amino acids that were found in the outer, mobile part of the cell wall may be part of proteins with such a function. *T*. *harzianum* might add an additional fortification layer against self-digestion by producing a diverse outer layer of polysaccharides and linkages that may not be recognized or that are not digestible by the secreted mixture of cell wall lytic enzymes (de Vries and Wessels, 1973).

### 3.4 FTIR results are in agreement with ssNMR on cell walls of T. harzianum T22

**Fig. 5:**
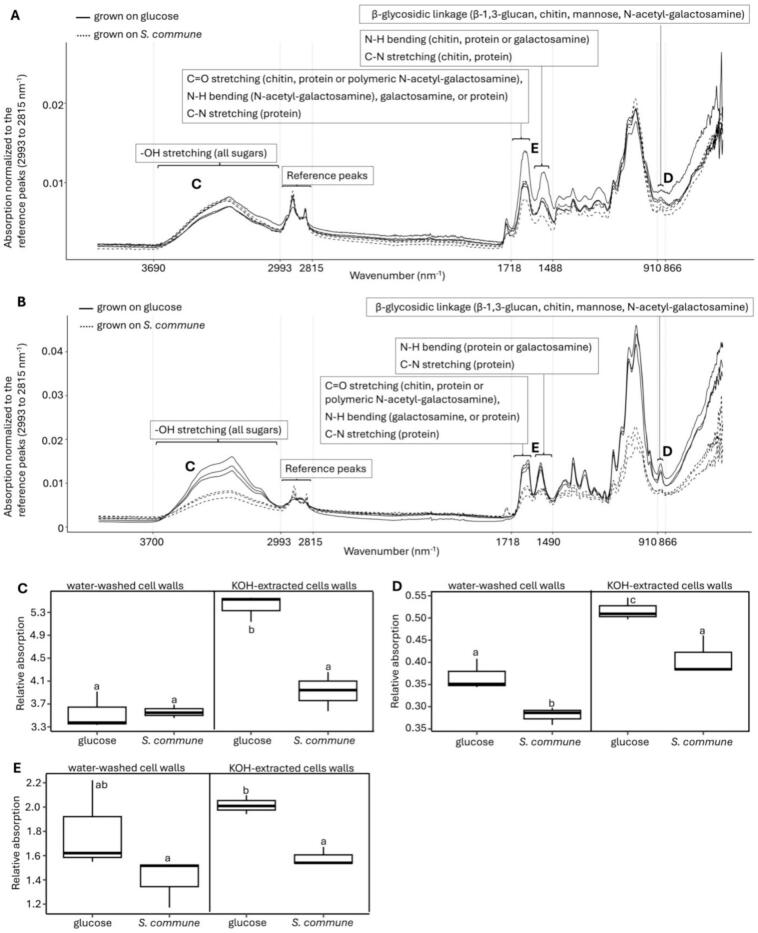
The effect of growth on glucose or water-washed *S. commune* cell walls on the *T. harzianum* T22 cell wall composition. FTIR spectra are shown of the water-washed (**A**) and the KOH-extracted (**B**) cell walls of *T. harzianum* T22. Relative absorption normalized to the reference peaks (2993 to 2815 nm^−1^) of cell wall samples at 3690 to 2993 nm^−1^ (**C**), 910 to 866 nm^−1^ (**D**) and at 1718 to 1488 nm^−1^ (**E**) comparing water-washed and KOH-extracted cell walls. Letters in the FTIR spectra (**A-B**) correspond to the boxplots (**C-E**). Absorption (**C-E**) was normalized to the absorption of the reference peaks at 2993 to 2815 nm^−1^. Letters in the boxplots (C-E) show significance (*p* ≤ 0.05) between growth on glucose and on water-washed *S. commune* cell walls.

FTIR was conducted to complement results obtained using ssNMR on water-extracted cell walls of *T*. *harzianum* T22 after growing the fungus on MM-N with glucose and on water-washed *S. commune* cell walls. The amount of polysaccharide in the cell wall was determined by normalizing the area underneath the peak at 3690 to 2993 nm^−1^ (-OH stretching) and at 910 to 866 nm^−1^ (β-glycosidic linkages) to the area underneath the reference peak at 2993 to 2815 nm^−1^ (Hong et al., 2021; Slavov et al., 2024) (Fig. 5C, D). The relative absorbances of -OH stretching of the water-extracted *T*. *harzianum* T22 cell walls grown on glucose and *S. commune* cell walls were similar. However, the absorbance of β-glycosidic linkages was 1.3-fold higher in the case of the cell walls isolated from the glucose culture. Next, absorption at 1718 to 1488 nm^−1^ by C=O stretching, N—H bending and 15d C—N stretching (Hong et al., 2021; Slavov et al., 2024; Stani et al., 2020) was used to analyze the amount of chitin, polymeric *N*-acetyl-galactosamine, galactosamine and amino acids and proteins in the cell wall (Fig. 5E). The normalized areas under the peaks showed no difference between the water-washed cell walls of *T*. *harzianum* T22 grown on glucose or on water-washed *S. commune* cell walls.

Previously, we have shown that KOH removes proteins, lipids as well as KOH-extractable polysaccharides from the water-washed cell walls (Ehren et al., 2020). This method was used to separate the protein signal from those of the amine containing poylsaccharides. The relative absorption at 1718 to 1488 nm^−1^ was ∼1.4-fold higher for KOH-extracted cell walls from cultures grown on glucose when compared to those grown on water-washed *S. commune* cell walls (Fig. 5E). This suggests that the *T*. *harzianum* T22 cell wall contains more chitin, polymeric *N*-acetyl-galactosamine and/or galactosamine after growth on glucose when compared to growth on *S. commune* cell walls. This notion is in line with the higher chitin signals found by ssNMR in the *T*. *harzianum* T22 cell walls isolated from glucose-grown cultures when compared to cultures grown on cell walls of *S. commune*. Also, relative absorption by -OH stretching groups and β-glycosidic linkages was ∼1.5-fold higher in KOH-extracted cell walls from glucose-grown cultures when compared to cultures grown on *S. commune* cell walls*.* Together, the FTIR results are in line with the ssNMR data that suggest that the cell wall of the glucose-grown *T*. *harzianum* T22 cultures is thicker than that of cultures grown on *S. commune* cell walls.

## 4 Discussion

**Fig. 6:**
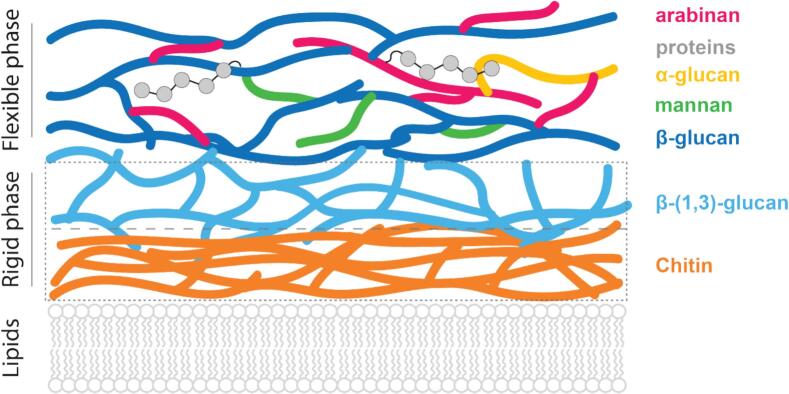
Organization of the *T*. *harzianum* T22 cell wall based on ssNMR data. This schematic model is partially adapted from the model presented in Ehren et al. (2020) The rigid cell wall consists of β-(1,4)-chitin (orange) and β-(1,3)-glucan (light blue). The flexible cell wall consists of arabinan (magenta), β-glucan (navy blue), α-glucan (yellow), and mannan (green) and proteins (grey orbs). The lipid signals that were detected in this study are probably from the plasma membrane underneath the chitin layer. (For interpretation of the references to colour in this figure legend, the reader is referred to the web version of this article.)

Combining our dipolar- and scalar-based solid-state NMR data (**Figs. 1 A** and **4 A**) as well as the analysis of the relative abundance of the different molecular species analyses (Figs. 3**B** and 4**B**) leads to the schematic model of the organization of the *T*. *harzianum* T22 cell wall shown in Fig. 6. In detail, the depicted architecture of the rigid cell wall phase of T22 relies on data from the ^1^H-detected relaxation (Lewandowski et al., 2011) and ^13^C-detected water-edited (Ader et al., 2008; Weingarth et al., 2012) ssNMR experiments. The ^13^C T_1ρ_ times of rigid β-(1,3)-glucan and chitin carbons (Fig. 2A) demonstrate that rigid β-(1,3)-glucan chains are much more dynamic and could be shorter than the chitin chains. In combination with our finding that rigid β-(1,3)-glucan is also more accessible to water (Fig. 2B), it seems likely that this layer is located closer to the cell wall exterior than the denser rigid chitin core that consists of longer polysaccharide chains. Such a notion would be in line with the work of Kang et al. (2018), who utilized information deduced from water-edited ssNMR experiments to postulate that β-(1,3)-glucan is located in the hydrated and mobile segment of the inner domain of *A. fumigatus* cell walls.

Comparison of the weighted ^13^C T_1ρ_ times between rigid cell wall components of *T*. *harzianum* T22 with those of *S. commune* H4-8 A (**Fig. S3**) yields high similarity between the rigid chitin of T22 and the rigid α-(1,3)-glucan of the *S. commune* cell walls from liquid shaken cultures (see **Tables S11** through **S14** for fitting parameters of cell wall polysaccharides isolated from *S. commune* liquid shaking cultures and static agar plate cultures similar to *T*. *harzianum* T22). In our model of *T*. *harzianum* T22, rigid chitin thus takes over the dominant role similar to that in the zygomycete *R*. *delemar* (Cheng et al., 2024) and in the case germinating conidia of *Aspergillus fumigatus* (Lamon et al., 2023), while rigid α-glucans (Jelsma and Kreger, 1978; Shetty et al., 2021) are prevalent in *S. commune* (Ehren et al., 2020) and *A. fumigatus* (Gautam et al., 2025; Kang et al., 2018) where the polysaccharide maintains an alkali-insoluble rigid inner cell wall core. The proposed localization of chitin in the inner core of the T22 cell wall is also congruent with the heightened vulnerability to self-degradation by cell-secreted chitinases (de Vries and Wessels, 1973) we possibly observe when *Trichoderma* is grown on *S. commune* cell walls.

## 5 Conclusions and perspectives

We have used ssNMR and FTIR methodology to determine the composition and architecture of the *T*. *harzianum* T22 cell wall. Importantly, our ssNMR analysis involved intact cell walls of *T*. *harzianum* without the need of extraction methods (Saravanakumar et al., 2021) that alter the structural, dynamical and possibly chemical composition of the samples. By combining carbon- and proton-detected ssNMR spectroscopy, we studied the dynamics and water-accessibility of major cell wall components and were able to deconvolute the organization of the rigid cell wall phase of T22. Our findings suggest that the polymer species in the *T*. *harzianum* cell wall are not influenced by differential growth conditions but that the relative abundance of rigid and flexible components is impacted. Growth on isolated *S. commune* cell wall material reduces chitin content in the rigid cell wall phase and depletes the abundance of flexible polysaccharides. Furthermore, we were able to shed light on the role of chitin in the *T*. *harzianum* cell wall and its potential susceptibility to self-digestion. Not just the complete absence of the digestion-sensitive α-(1,3)-glucan species in the rigid cell wall core but also the presence of a diverse flexible outer cell wall layer could serve as protection mechanism against self-digestion and thus play a vital role in the functioning of *T*. *harzianum* as a (myco)parasite and possibly in other (un)related mycoparasitic species.

Our results reported here provide further support for the use of ssNMR to study other complex fungal systems that are of vital importance in agricultural and food applications as well as in human infections. Such information can be also used to infer the behavior and capacity to adaptation of the fungal systems that play an essential role in maintaining a pressured ecological balance. The combination of techniques described here allow for atomic level studies providing chemical as well as quantitative insight into the molecular composition of the fungal cell wall. Notably, our quantification of the different molecular species yields relative measures among the rigid and dynamic species detected. For a precise absolute quantification, additional experiments need to be designed that probe NMR signals stemming from the entire NMR sample irrespective of the dynamic properties. In addition, sufficient spectral resolution and sensivity must be estalished. For the latter reason, we used isotope (^13^C,^15^N) labeled samples which can represent a complication for ssNMR in case labelling protocols such as described in section 2.2. are not available. Dynamic nuclear polarization (DNP) has been used to greatly enhance ssNMR signal intensities of native fungal cell walls (Zhao et al., 2020). Alternatively, ^1^H-detected ssNMR (Bahri et al., 2023; Safeer et al., 2023) alleviates this limitation without the need to conduct experiments in frozen conditions.

In addition, we have shown how ssNMR methods can be used to probe dynamic properties as well as the surface exposure of the different molecular species at atomic resolution. ^1^H-detected experiments are already available for the measurements of slow dynamics (Chill et al., 2006; Lewandowski, 2013; Lewandowski et al., 2011) that reduce the need for isotope labelling and increase spectral sensivity. For the same purpose, we have recently reported ssNMR methods that probe water exposure in fungal cell walls using ^1^H detection (Bishoyi et al., 2025) that may be applicable to fungal cell wall systems that contain ^13^C/^15^N nuclei at natural abundance.

## CRediT authorship contribution statement

**A.A. Safeer:** Writing – review & editing, Writing – original draft, Visualization, Validation, Methodology, Investigation, Formal analysis, Data curation, Conceptualization. **F.E.L. Kleijburg:** Writing – review & editing, Writing – original draft, Visualization, Validation, Methodology, Investigation, Formal analysis, Data curation, Conceptualization. **H.A.B. Wösten:** Writing – review & editing, Writing – original draft, Supervision, Project administration, Funding acquisition, Conceptualization. **M. Baldus:** Writing – review & editing, Writing – original draft, Supervision, Project administration, Funding acquisition, Conceptualization.

## Declaration of competing interest

The authors declare that they have no known competing financial interests or personal relationships that could have appeared to influence the work reported in this paper.

## Data Availability

Data will be made available on request.
