# Supplementary material for: Solid-state NMR spectroscopy reveals unique properties of Trichoderma harzianum cell wall components
Source: Cell Surf. 2025 Oct 11;14:100156. doi: 10.1016/j.tcsw.2025.100156 (PMC12550585; doi:10.1016/j.tcsw.2025.100156)
Supplement: Supplementary material [file mmc1.docx]

**Supporting Information**

***Solid-State NMR experimental data analysis***

NMRFAM-SPARKY(Lee et al., 2015) was used to create figures. Assignments for rigid and flexible cell wall polysaccharides are found in **Tables S1, S2, S7** and **S8**. For all shown spectra and assignment tables, the ^1^H dimension was referenced to the water peak at 4.7 ppm, and the ^13^C dimension calibrated using an external adamantane reference. For the analysis of ^13^C-T_1ρ_ 2D series, spectra were processed with the QSINE window function and shifted square sine bell (SSB) of 2 in the ^1^H dimension. Peaks were then integrated and integrals were fit by double-exponential regression. To determine the water-edited buildup times, 1D spectra were processed using 50 Hz line EM broadening and peak heights were fit with mono-exponentials. All curve fitting and half-time determination was performed using GraphPad Prism 9.5.1. The resulting fit parameters and output values are shown in **Table S4**, **S5**, **S11**, **S12**, **S13** and **S14**.

The relative abundance (RA) analysis(Chakraborty et al., 2021; Safeer et al., 2023) of rigid cell wall species was performed by integrating a single representative, isolated (resolved) peak per polysaccharide type in the acquired dipolar-based 2D ^1^H-^13^C spectra. The integration function in TopSpin 4.1.0 (Bruker BioSpin) was used to obtain peak volumes (*V*) (see **Table S3**). The assumption was made that the *V* for every CH pair of a polysaccharide type present in a 2D ^1^H-^13^C spectrum would be roughly equivalent at the applied CP contact times. The RA per rigid polysaccharide type was calculated using the following formula:

$$\mathrm{RA} (\%)=\frac{V_{polysaccharide}*expected CH pairs}{\sum(V_{polysaccharide}*expected CH pairs)+V_{bulk lipid}}*100$$

where the expected CH pairs for glucose-based and glucosamine polymers were respectively 6 and 7, and the bulk lipid peak at ~35 ppm was assumed to represent the total ensemble of lipid CH pairs present in dipolar-based 2D ^1^H-^13^C correlation spectra. The RA per cell wall species is reported in **Table S6**. Error was based on the signal-to-noise determined per integral using the sino2d function in TopSpin 4.1.0 (Bruker BioSpin).

The relative abundance analysis of all flexible cell wall polymers (lipids, polysaccharides, and polypeptides) (Safeer et al., 2023) was performed by considering the cumulative peak intensity per cell wall polymer in the acquired scalar-based 2D ^1^H-^13^C spectra. The relative abundance per flexible cell wall polysaccharide was determined by considering the peaks in the carbohydrate C1 region (between 90-110 ppm in the ^13^C dimension) as representative for each polysaccharide type. Peak intensities were used due to limited spectral crowding and high similarity in ^1^H linewidth in the C1 region. Error was based on the signal-to-noise of peak intensities. C1 peak intensities are summarized in **Table S9**.


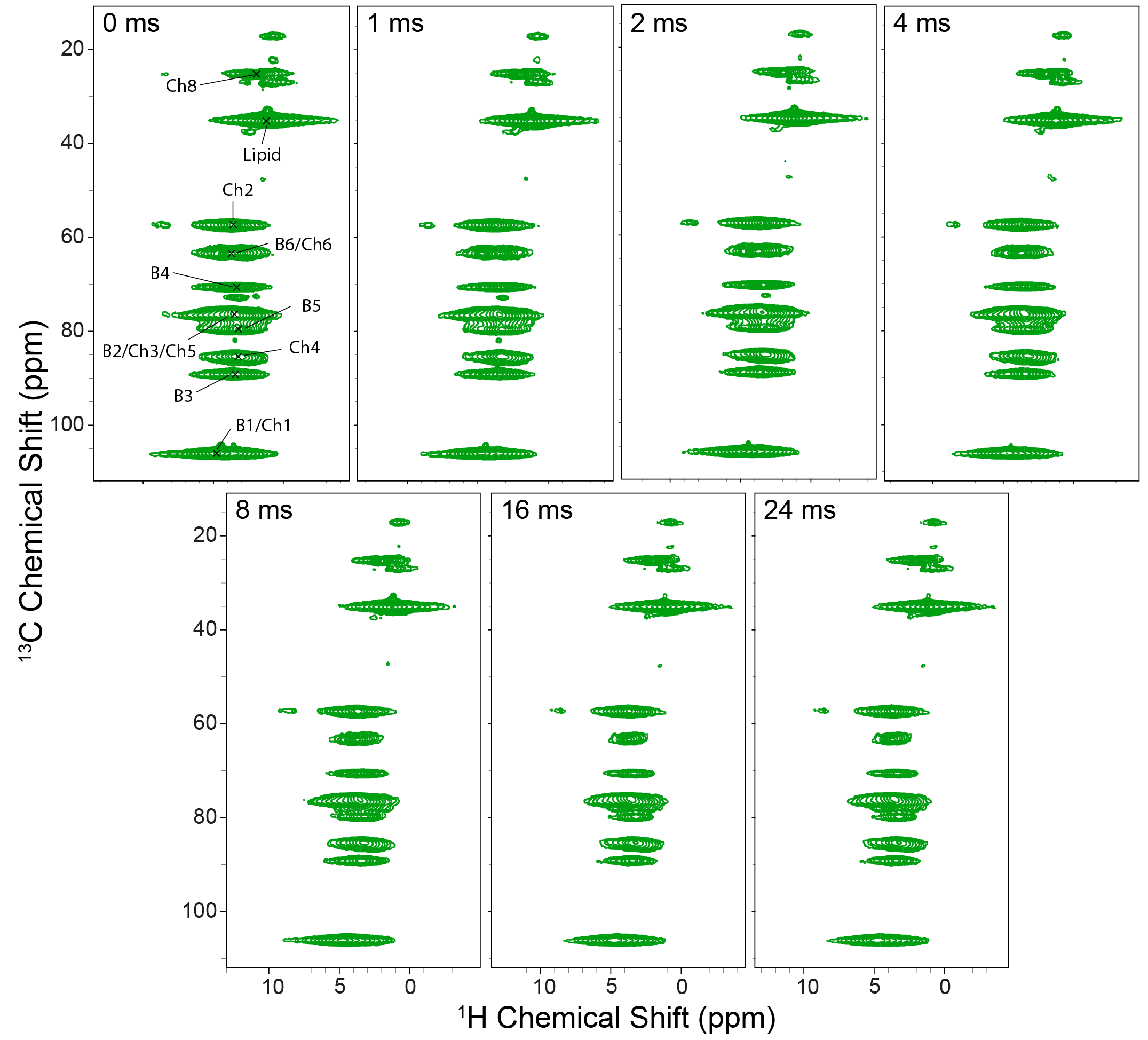


**Figure S1.** 2D dipolar ^1^H-^13^C correlation spectra of water-washed *T*. *harzianum* T22 cell walls from the T_1ρ_ relaxation series.


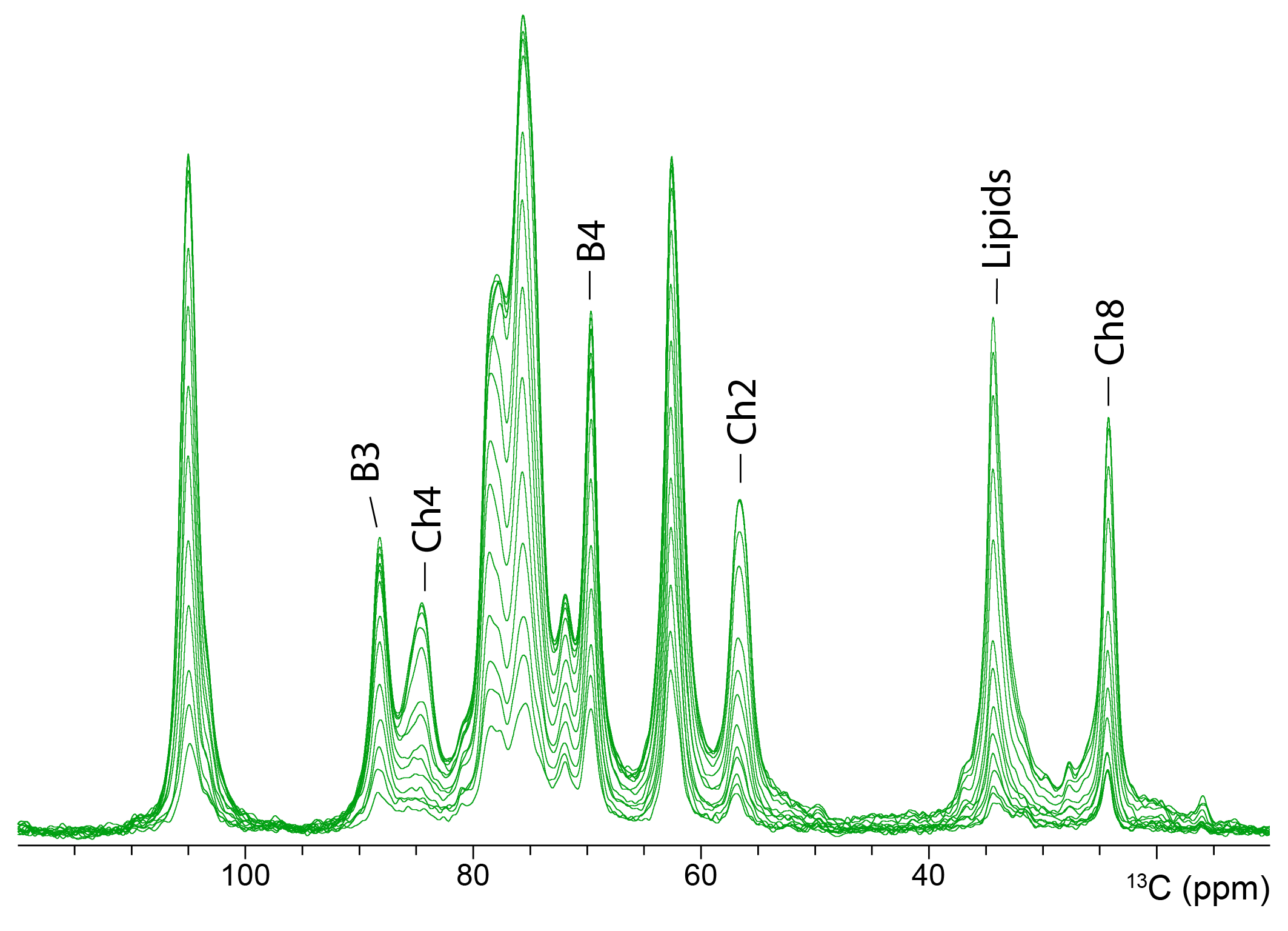


**Figure S2.** ^13^C-detected 1D water-edited CP experiment series of water-washed *T*. *harzianum* T22 cell walls with increasing mixing time (0 - 64 ms) yielding more signal.


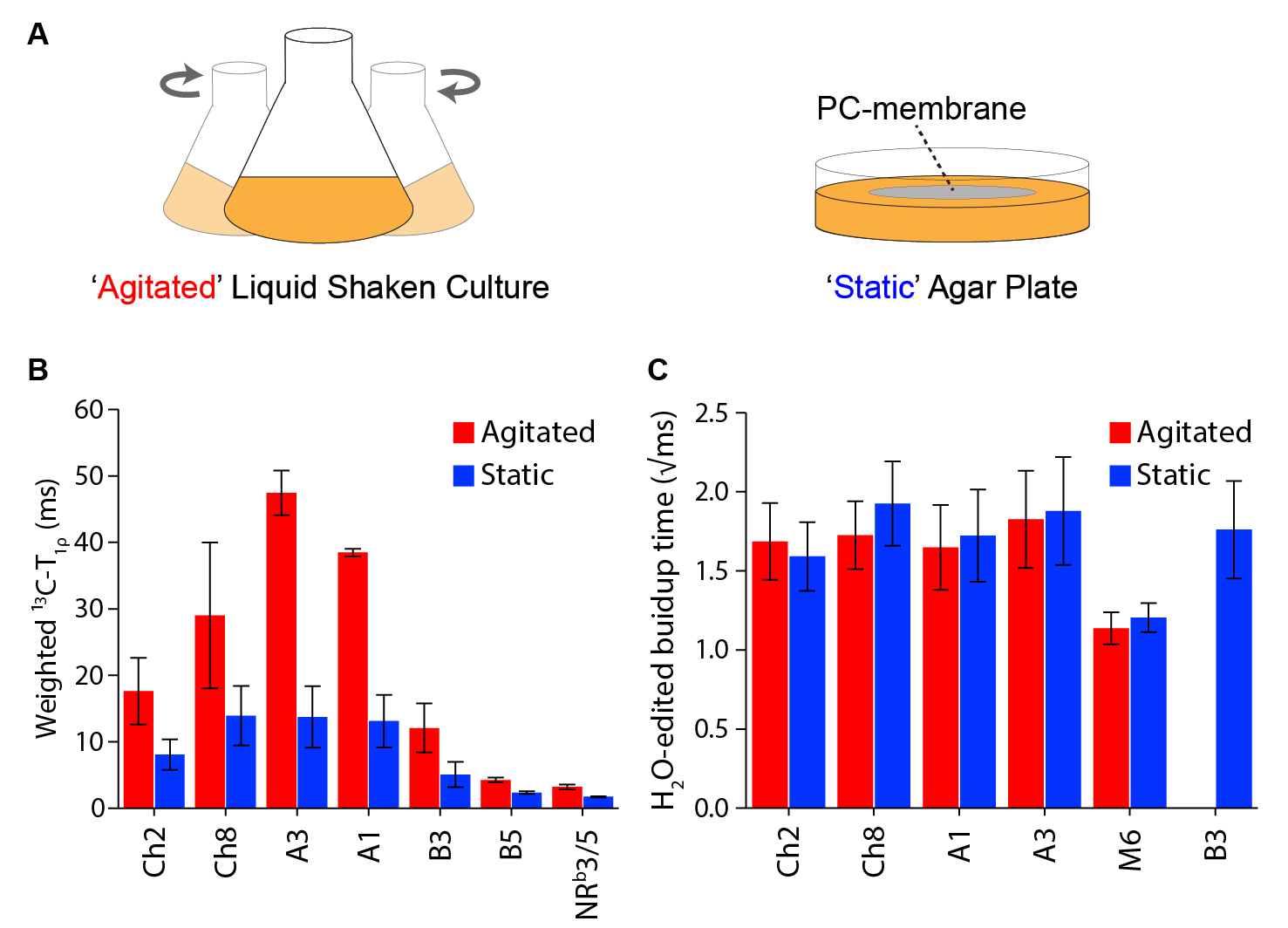


**Figure S3.** *S. commune* cell wall dynamics and water-accessibility. **A)** Schematic representations of the tested *S*. *commune* growth methods with MM-N.(Kleijburg et al., 2023) **B)** Weighted T_1ρ_ relaxation times for water-washed *S*. *commune* cell walls **B)** Signal buildup times determined from a series of 1D water-edited ^13^C-detected spectra gives a measure of water-accessibility for the rigid components of water-washed *S*. *commune* cell walls. Tested polysaccharides are α-(1,3)-glucan (A), β-(1,3)-glucan (B), chitin (Ch) and non-reducing β-glucan (NR^b^) (Ehren et al., 2020; Safeer et al., 2023). Errors in peak intensities were based on the spectral noise level obtained from POKY from NMRFAM-Sparky (Lee et al., 2015).


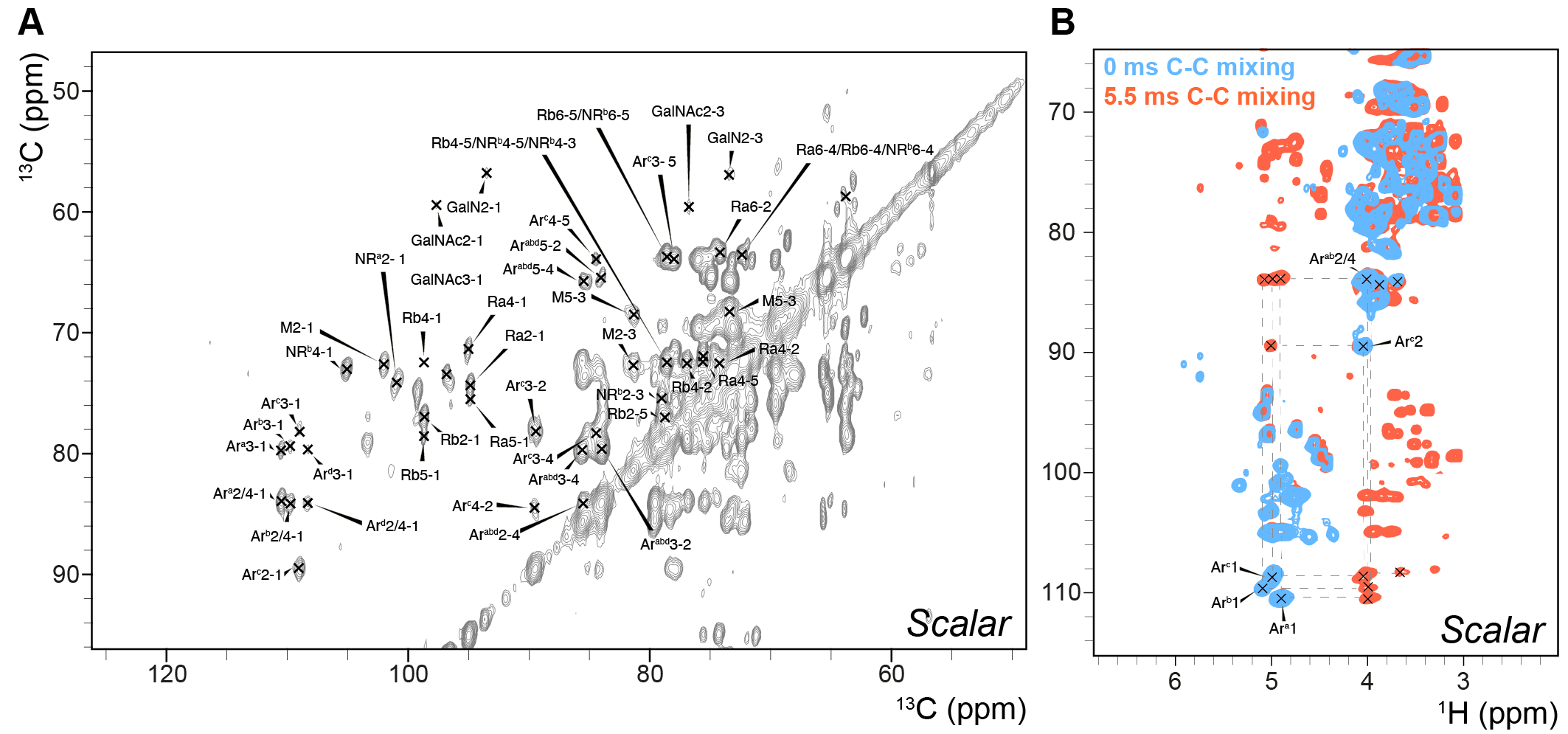


**Figure S4.** Scalar assignment of the *T*. *harzianum* T22 mobile cell wall via scalar-based ssNMR experiments. Scalar-based ^1^H-^13^C 2D correlation spectra with (red) and without (light blue) 5.5 ms of DIPSI C-C mixing.


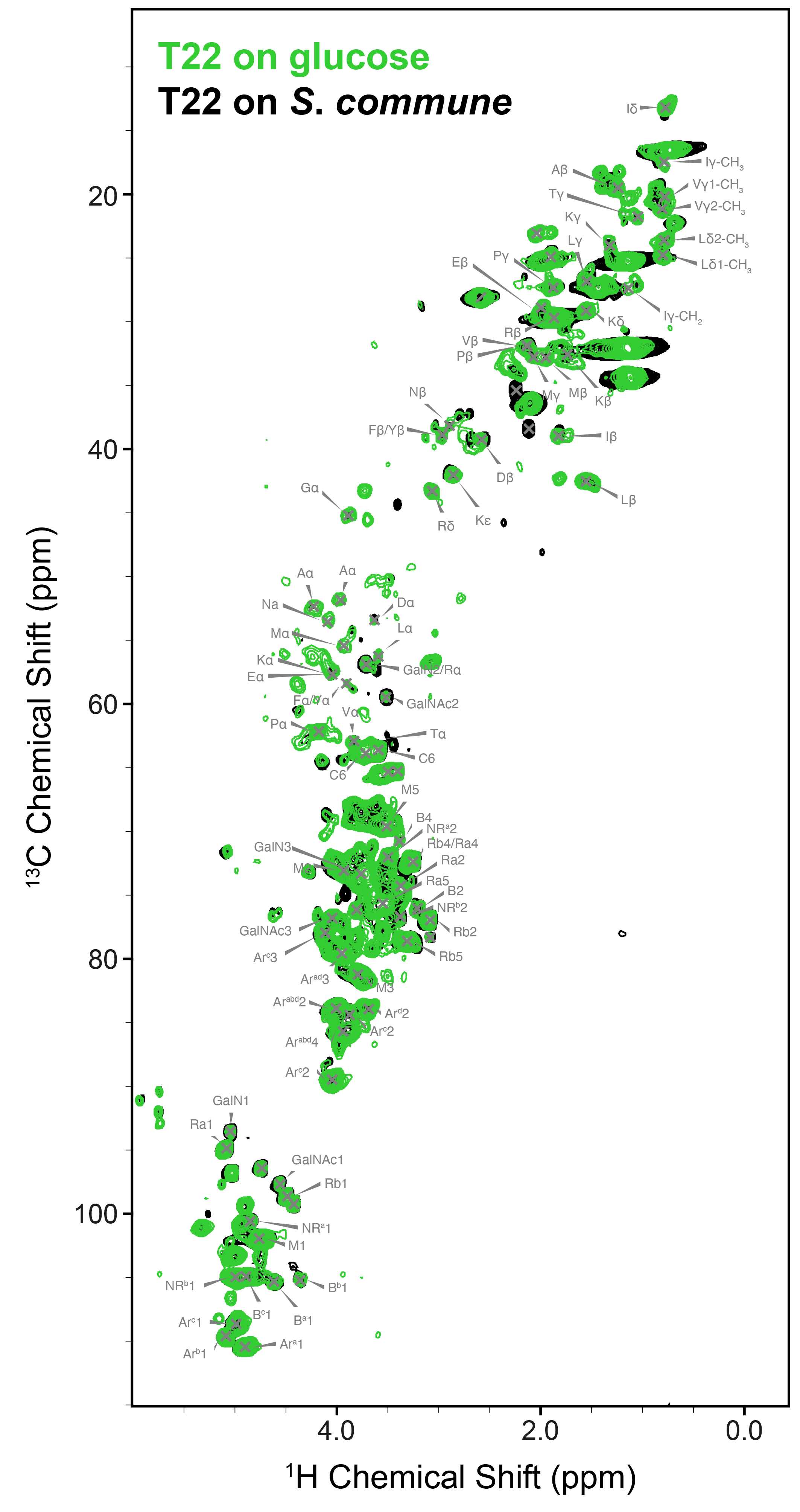


**Figure S5.** 2D scalar ^1^H-^13^C correlation spectrum of water-washed cell walls of *T*. *harzianum* T22 grown on glucose overlaid on water-washed cell walls from T22 grown on U-^13^C,^15^N-labeled *S*. *commune* cell wall material.


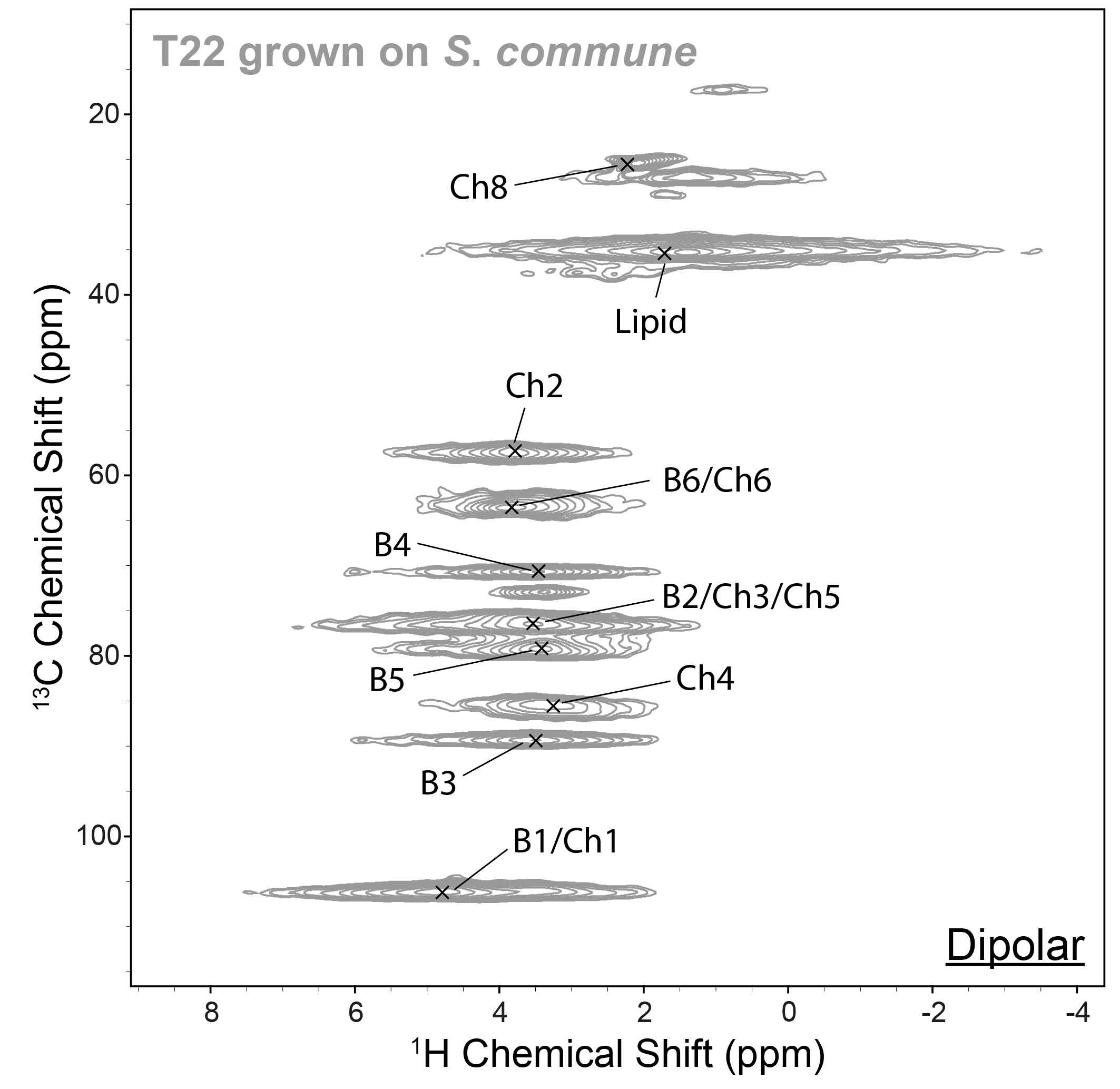


**Figure S6.** 2D dipolar ^1^H-^13^C correlation spectrum of cell wall material isolated from *T*. *harzianum* T22 grown on U-^13^C,^15^N-labeled *S*. *commune* cell wall material.


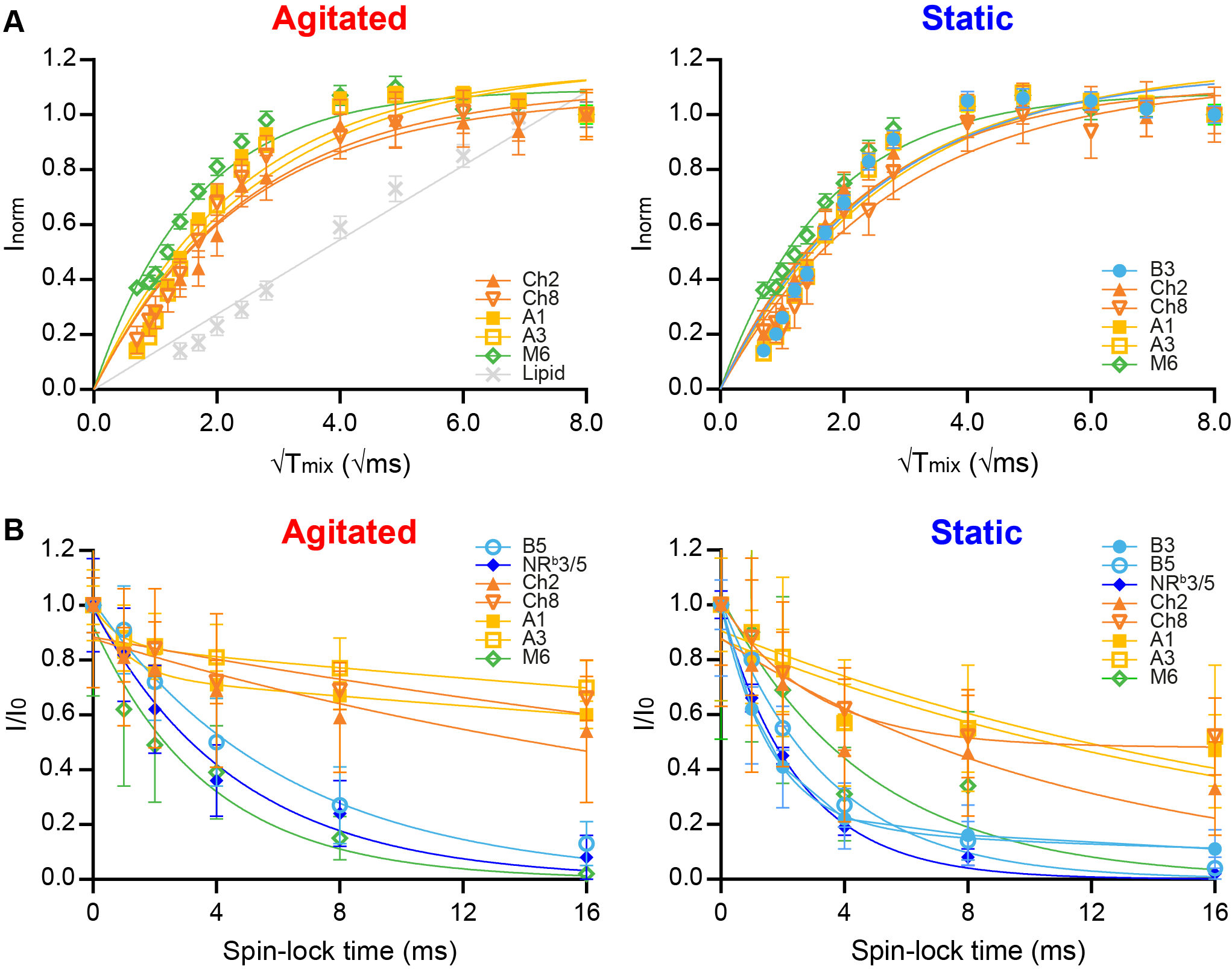
**Figure S7.** T_1ρ_ relaxation signal decay and water-edited buildup curves of *S*. *commune* cell wall material isolated from mycelium grown on MM-N in liquid shaken (left) and static (right) conditions.(Kleijburg et al., 2023) **A)** Integrals of peaks from dipolar 2D ^1^H-^13^C correlation spectra were normalized and ^13^C-T_1ρ_ relaxation decays were fit with a bi-exponential decay. **B)** Signal intensities from ^13^C-detected 1D water-edited CP experiments were normalized and fit with a mono-exponential decay. Errors in peak intensities were based on the spectral noise level obtained from POKY from NMRFAM-Sparky (Lee et al., 2015).


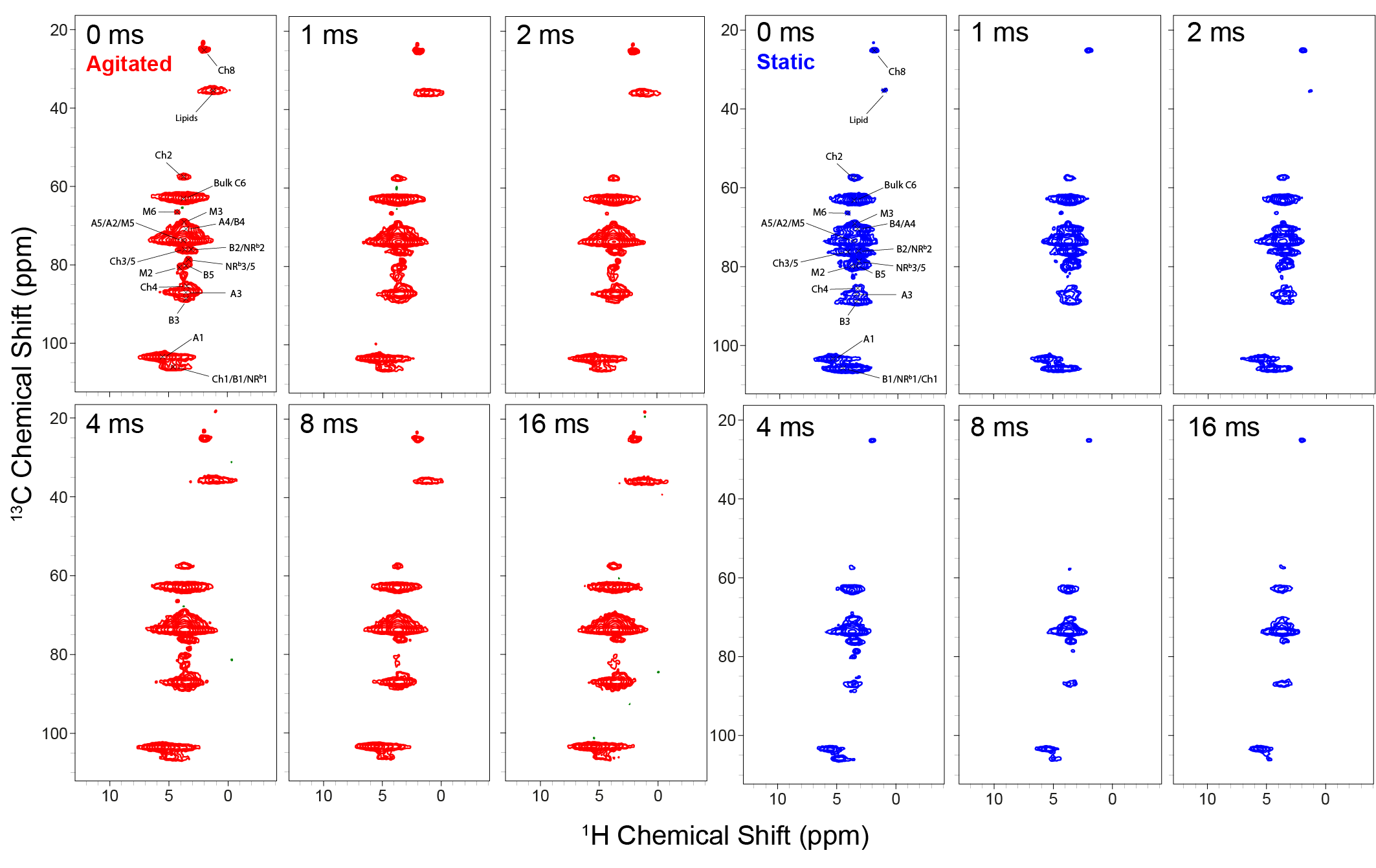
 **Figure S8.** 2D dipolar ^1^H-^13^C correlation spectra from the T_1ρ_ relaxation series of water-washed cell walls isolated from *S*. *commune* mycelium grown under liquid shaken (red) and static (blue) conditions on MM-N (Kleijburg, 2024; Kleijburg et al., 2023).


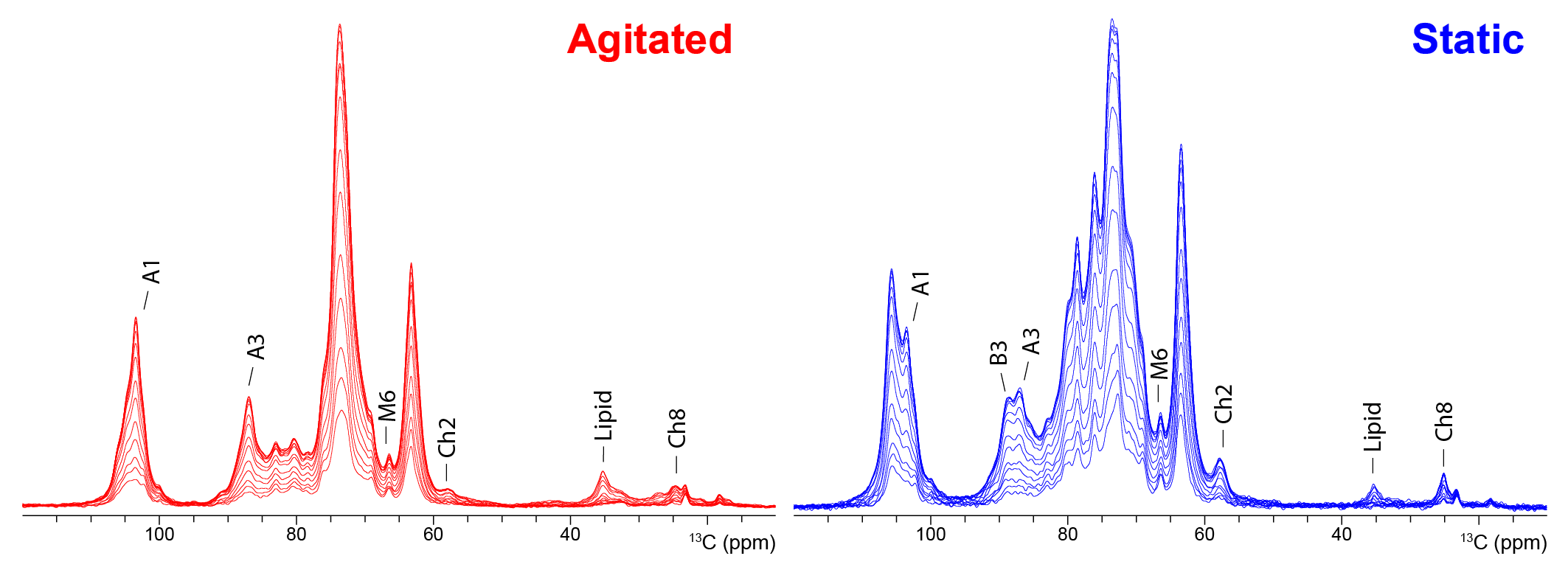


**Figure S9.** ^13^C-detected 1D water-edited CP experiment series with increasing mixing time (0 - 64 ms) of *S*. *commune* cell wall material isolated from mycelium grown under liquid shaken (red) and static (blue) conditions on MM-N (Kleijburg, 2024).


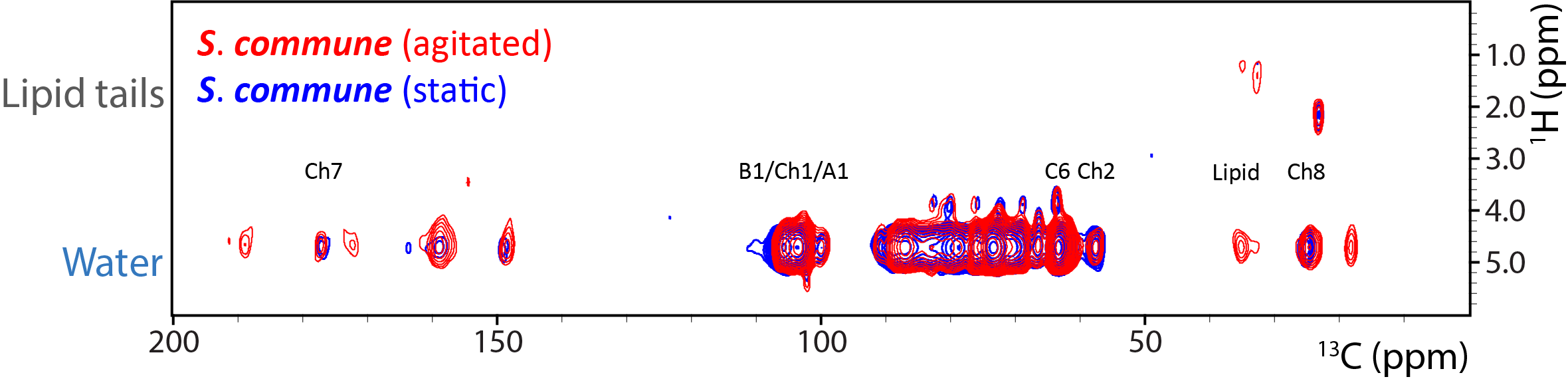


**Figure S10.** Topology of the *S*. *commune* cell wall isolated from mycelium grown under liquid shaken (red) and static (blue) conditions on MM-N (Kleijburg, 2024), as probed by dipolar-based water-edited ^13^C-^1^H 2D correlation spectrum using a mixing time of 5 ms.

**Table S1.** ssNMR acquisition and processing parameters.

| **Sample** | **Experiment** | **scans / dummy scans** | **90º r.f. power (kHz)** | **Direct / indirect acqu time (ms)** | **MAS (kHz)** | **Set temperature (K)** | **Window function (SSB)** |
| --- | --- | --- | --- | --- | --- | --- | --- |
| T22 on glucose | Scalar HSQC | 32 / 32 | 139 (**^1^**H) / 99 (^13^C) | 29.3 / 14.4 | 54 | 258 | QSINE (2.5) |
|  | Dipolar hCH | 32 / 16 | 139 (**^1^**H) / 90 (^13^C) | 9.24 / 8.74 | 54 | 258 | QSINE (4) |
|  | CP-DQSQ | 128 / 32 | 66 (**^1^**H) / 51 (^13^C) | 7.47 / 2.37 | 8 | 265 | QSINE (2.5) |
|  | INEPT-TOBSY | 96 / 32 | 66 (**^1^**H) / 51 (^13^C) | 23.3 / 5.68 | 13 | 260 | QSINE (2) |
|  | T2-HC | 128 / 4 | 69 (**^1^**H) / 50 (^13^C) | 5.76 / 2.97 | 15 | 275 | QSINE (3 / 2) |
| T22 on *S*. *commune* | Scalar HSQC | 128 / 32 | 178 (**^1^**H) / 90 (^13^C) | 29.3 / 14.4 | 54 | 258 | QSINE (2.5) |
|  | Dipolar hCH | 64 /16 | 178 (**^1^**H) / 90 (^13^C) | 9.24 / 8.74 | 54 | 258 | QSINE (4) |
| *S*. *commune* | CP-DQSQ | 128 / 32 | 69 (**^1^**H) / 53 (^13^C) | 7.47 / 2.37 | 8 | 265 | QSINE (2.5) |

**Table S2.** Resonance assignments of signals from the 2D dipolar ^1^H-^13^C correlation experiment of water-washed *T*. *harzianum* T22 cell walls.

| **Molecular species** | **C1 (ppm)**  **H1 (ppm)** | **C2 (ppm)**  **H2 (ppm)** | **C3 (ppm)**  **H3 (ppm)** | **C4 (ppm)**  **H4 (ppm)** | **C5 (ppm)**  **H5 (ppm)** | **C6 (ppm)**  **H6 (ppm)** | **C8 (ppm)**  **H8 (ppm)** |
| --- | --- | --- | --- | --- | --- | --- | --- |
| Chitin | 106.126  4.784 | 57.425  3.703 | 76.622  3.449 | 85.381  3.313 | 76.622  3.449 | 63.549  3.735 | 25.343  1.983 |
| β-(1,3)-glucan | 106.126  4.784 | 76.622  3.449 | 89.239  3.443 | 70.624  3.39 | 79.651  3.301 | 63.549  3.735 |  |

**Table S3.** Resonance assignments of signals from the 2D ^13^C-^13^C Double-Quantum Single-Quantum experiment of water-washed *T*. *harzianum* T22 cell walls.

| **Molecular species** | **C1 (ppm)** | **C2 (ppm)** | **C3 (ppm)** | **C4 (ppm)** | **C5 (ppm)** | **C6 (ppm)** | **C7 (ppm)** | **C8 (ppm)** |
| --- | --- | --- | --- | --- | --- | --- | --- | --- |
| Chitin | 106.821 | 56.558 | 75.397 | 85.117 | 75.443 | 62.584 | - | - |
| β-(1,3)-glucan | 105.758 | 76.625 | 88.939 | 70.416 | 79.423 | 63.425 |  |  |

**Table S4.** Peak volumes of signals from the 2D dipolar ^1^H-^13^C correlation spectrum of the water-washed *T*. *harzianum* T22 cell walls.

|  | **T22 on glucose** | | **T22 on SC** | |
| --- | --- | --- | --- | --- |
| **Assignment** | **Peak volume** | **SNR** | **Peak volume** | **SNR** |
| Ch8 | 2.28·10^9^ | 159 | 1.34·10^9^ | 66.7 |
| Lipid | 4.14·10^9^ | 101 | 5.75·10^9^ | 91.7 |
| Ch2 | 2.44·10^9^ | 70.5 | 1.72·10^9^ | 47.3 |
| C6 | 3.37·10^9^ | 108 | 1.82·10^9^ | 45.1 |
| B4 | 2.21·10^9^ | 119 | 1.83·10^9^ | 67.4 |
| Ch3,5/B2 | 6.36·10^9^ | 112 | 4.75·10^9^ | 72.6 |
| B5 | 2.85·10^9^ | 68.8 | 2.58·10^9^ | 61.4 |
| Ch4 | 1.81·10^9^ | 30.1 | 1.04·10^9^ | 16.8 |
| B3 | 1.67·10^9^ | 43.9 | 1.37·10^9^ | 35.2 |
| Ch1/B1 | 4.70·10^9^ | 93.6 | 3.56·10^9^ | 63.9 |

List of abbreviations:

B: β-(1,3)-glucan
Ch: chitin

Numbers represent saccharide carbons: C1, C2, C3, C3, C4, C5, C6, C7 and C8

**Table S5.** Output parameters from the fitting of the T_1ρ_ signal decay of water-washed *T*. *harzianum* T22 cell walls.

|  | **B3** | **B4** | **Ch2** | **Ch4** | **Ch8** | **Lipid** |
| --- | --- | --- | --- | --- | --- | --- |
| p-value | 0.0048 | 0.0185 | 0.0212 | 0.0002 | 0.0018 | 0.0187 |
| PercentFast | 0.24 | 0.25 | 0.12 | 0.10 | 0.21 | 0.36 |
| PercentFast error | 0.03 | 0.06 | 0.02 | 0.00 | 0.01 | 0.06 |
| t½ slow (ms) | 13.4 | 11.6 | 54.8 | 52.3 | 76.4 | 54.8 |
| t½ slow error (ms) | 0.80 | 1.11 | 5.01 | 0.77 | 6.84 | 20.1 |
| t½ fast (ms) | 1.10 | 1.44 | 1.26 | 0.73 | 0.21 | 1.46 |
| t½ fast error (ms) | 0.24 | 0.47 | 0.48 | 0.06 | 0.13 | 0.51 |
| R-squared | 0.9991 | 0.9987 | 0.9957 | 0.9998 | 0.9949 | 0.9844 |
| Sum of Squares | 4.58·10^-4^ | 7.74·10^-4^ | 5.05·10^-4^ | 2.07·10^-5^ | 4.76·10^-4^ | 3.65·10^-3^ |
| Sy.x | 1.24·10^-2^ | 1.61·10^-2^ | 1.30·10^-2^ | 2.63·10^-3^ | 1.26·10^-2^ | 3.49·10^-2^ |

**Table S6.** Output parameters from the fitting of signal buildup during the water-edited 1D mixing time series of water-washed *T*. *harzianum* T22 cell walls.

|  | **B3** | **B4** | **Ch2** | **Ch4** | **Ch8** |
| --- | --- | --- | --- | --- | --- |
| t½ (ms) | 1.301 | 1.149 | 4.29 | 4.553 | 5.937 |
| t½ error (ms) | 0.2688 | 0.1682 | 0.856 | 1.072 | 1.69 |
| R-squared | 0.8402 | 0.8904 | 0.9724 | 0.9667 | 0.9699 |
| Sum of Squares | 0.2775 | 0.1417 | 0.04324 | 0.05417 | 0.04813 |
| Sy.x | 0.1521 | 0.1087 | 0.06003 | 0.06719 | 0.06333 |

**Table S7.** Assignments of resonances in the 2D scalar ^1^H-^13^C correlation spectra of water-washed *T*. *harzianum* T22 cell walls.

| **Molecular species** | **C1 (ppm)**  **H1 (ppm)** | **C2 (ppm)**  **H2 (ppm)** | **C3 (ppm)**  **H3 (ppm)** | **C4 (ppm)**  **H4 (ppm)** | **C5 (ppm)**  **H5 (ppm)** |
| --- | --- | --- | --- | --- | --- |
| Ar^a^ | 110.478  4.899 | 83.895  4.009 | 79.538  3.948 | 85.685  3.939 |  |
| Ar^b^ | 109.619  5.09 | 83.895  4.009 | 79.538  3.948 | 85.685  3.939 |  |
| Ar^c^ | 108.669  4.991 | 89.483  4.045 | 78.379  3.826 | 85.685  3.939 |  |
| B^a^ | 105.305  4.616 | 76.019  3.208 |  | 70.78  3.385 |  |
| B^b^ | 105.198  4.36 | 76.019  3.208 |  | 70.78  3.385 |  |
| B^c^ | 104.897  4.893 | 76.019  3.208 |  | 70.78  3.385 |  |
| Rb | 98.654  4.485 | 76.928  3.085 |  | 72.357  3.253 | 78.585  3.312 |
| Ra | 94.882  5.081 | 74.301  3.372 |  | 72.357  3.253 | 75.618  3.55 |
| NR^b^ | 104.991  4.995 | 76.694  3.379 |  |  |  |
| NR^a^ | 100.57  4.85 | 72.023  3.496 |  |  |  |
| GalN | 93.573  5.044 | 56.863  3.713 | 73.332  3.764 |  |  |
| GalNAc | 97.684  4.556 | 59.491  3.518 | 76.801  4.047 |  |  |
| M | 101.971  4.762 | 73.051  3.929 | 81.244  3.793 |  | 69.371  3.42 |

List of abbreviations:

Ar^a,b,c^: Arabinan

B^a,b,c^: β-(1,3)-glucan
GalNAc: N-acetyl galactosamine containing polysaccharide

GalN: galactosamine containing polysaccharide

M: mannan
NR^a^: non reducing end α-(1,3)-glucan

NR^b^: non reducing end β-(1,3)-glucan
Ra: reducing end α-(1,3)-glucan
Rb: reducing end β-(1,3)-glucan

**Table S8.** Assignments of resonances from the 2D ^13^C-^13^C INEPT-TOBSY spectrum of water-washed *T*. *harzianum* T22 cell walls.

| **Molecular species** | **C1 (ppm)** | **C2 (ppm)** | **C3 (ppm)** | **C4 (ppm)** | **C5 (ppm)** | **C6 (ppm)** |
| --- | --- | --- | --- | --- | --- | --- |
| Ar^a^ | 110.53 | 83.935 | 79.722 | 83.935 | 65.704 |  |
| Ar^b^ | 109.773 | 84.11 | 79.389 | 84.11 | 65.704 |  |
| Ar^c^ | 109.06 | 89.47 | 78.178 | 84.476 | 63.917 |  |
| Ar^d^ | 108.295 | 84.107 | 79.659 | 84.107 | 65.704 |  |
| NR^b^ | 105.081 | 75.425 | 79.008 | 73.026 |  | 63.687 |
| NR^a^ | 100.949 | 74.108 |  | 73.737 |  | 63.361 |
| Rb | 98.696 | 76.952 |  | 72.39 | 78.545 | 63.687 |
| Ra | 95.024 | 74.357 |  | 71.335 | 75.619 | 63.529 |
| M | 102.03 | 72.684 | 81.331 |  | 68.507 |  |

List of abbreviations:

Ar^a,b,c^: Arabinan

GalNAc: N-acetyl galactosamine containing polysaccharide

GalN: galactosamine containing polysaccharide

M: mannan
NR^a^: non reducing end α-(1,3)-glucan

NR^b^: non reducing end β-(1,3)-glucan
Ra: reducing end α-(1,3)-glucan
Rb: reducing end β-(1,3)-glucan

**Table S9.** Relative abundance of rigid polymers in water-washed *T*. *harzianum* T22 cell walls.

|  | **T22 on glucose** | | | **T22 on SC** | | |
| --- | --- | --- | --- | --- | --- | --- |
| **Resolved species** | **Rel. volume** | **Rel. contribution** | **Error** | **Rel. volume** | **Rel. contribution** | **Error** |
| Ch(2) | 7 · 2.44·10^9^ | 50% | 0.2% | 7 · 1.72·10^9^ | 42% | 0.3% |
| B(4) | 6 · 2.21·10^9^ | 38% | 0.4% | 6 · 1.83·10^9^ | 38% | 0.3% |
| Lipid | 4.14·10^9^ | 12% | 1% | 5.75·10^9^ | 20% | 1% |
| **Total** | 3.45·10^10^ | 100% |  | 2.88·10^10^ | 100% |  |

List of abbreviations:

B: β-(1,3)-glucan
Ch: chitin

Numbers represent saccharide carbons C1, C2, C3, C3, C4, C5, C6, C7 and C8

**Table S10.** C1 peak intensities of flexible polysaccharide species from 2D scalar ^1^H-^13^C correlation spectra of water-washed *T*. *harzianum* T22 cell walls.

|  | **T22 on glucose** | | **T22 on *S*. *commune*** | |
| --- | --- | --- | --- | --- |
| **Polysaccharide species** | **Peak intensity** | **SNR** | **Peak intensity** | **SNR** |
| Ar^a,b,c^ | 1.45·10^8^ | 141 | 2.22·10^7^ | 21.4 |
| β-(1,3)-glucan | 1.38·10^8^ | 19.9 | 8.39·10^7^ | 67.9 |
| Rb | 6.79·10^7^ | 412 | 5.77·10^7^ | 346 |
| B^a,b,c^ | 1.66·10^7^ | 11.5 | 1.44·10^7^ | 5.32 |
| NR^b^ | 5.06·10^7^ | 307 | 1.18·10^7^ | 70.9 |
| α-(1,3)-glucan | 5.48·10^8^ | 25.7 | 3.86·10^7^ | 70.9 |
| Ra | 5.06·10^7^ | 307 | 3.54·10^7^ | 212 |
| NR^a^ | 4.26·10^7^ | 25.8 | 3.18·10^7^ | 19.1 |
| Mannan | 5.52·10^7^ | 335 | 1.31·10^7^ | 78.4 |
| GalN(Ac) | 1.51·10^7^ | 32.1 | 4.31·10^7^ | 89.8 |
| GalNAc | 7.06·10^6^ | 42.8 | 1.93·10^7^ | 115 |
| GalN | 8.00·10^6^ | 48.6 | 2.39·10^7^ | 143 |
| Rest / other | 1.55·10^8^ | 6.07 | 7.55·10^7^ | 2.52 |
| Total | 5.67·10^8^ |  | 2.67·10^8^ |  |

List of abbreviations:

Ar^a,b,c^: Arabinan

B^a,b,c^: β-(1,3)-glucan
GalNAc: N-acetyl galactosamine containing polysaccharide

GalN: galactosamine containing polysaccharide

M: mannan
NR^a^: non reducing end α-(1,3)-glucan

NR^b^: non reducing end β-(1,3)-glucan
Ra: reducing end α-(1,3)-glucan
Rb: reducing end β-(1,3)-glucan

**Table S11.** Output parameters for the fitting of T_1ρ_ signal decay for water-washed cell wall material from *S*. *commune* MM-N liquid shaken cultures.

|  | **Ch2** | **Ch8** | **A1** | **A3** | **B5** | **NRb3/5** |
| --- | --- | --- | --- | --- | --- | --- |
| Preferred model | One phase decay | One phase decay | Two phase decay | Two phase decay | One phase decay | One phase decay |
| p-value | 0.065 | 0.1573 | <0.0001 | 0.0061 | 0.5559 | 0.3972 |
| t½ (ms) | 17.62 | 29.01 | - | - | 4.285 | 3.229 |
| t½ error (ms) | 5.027 | 10.98 | - | - | 0.3603 | 0.3605 |
| PercentFast | - | - | 25.51 | 14.63 | - | - |
| PercentFast error | - | - | 0.1691 | 0.8807 | - | - |
| t½ slow (ms) | - | - | 51.39 | 55.49 | - | - |
| t½ slow error (ms) | - | - | 0.7646 | 3.941 | - | - |
| t½ fast (ms) | - | - | 0.6991 | 0.5412 | - | - |
| t½ fast error (ms) | - | - | 0.0108 | 0.08465 | - | - |
| R squared | 0.7887 | 0.6578 | 1 | 0.9987 | 0.9893 | 0.9827 |
| Sum of Squares | 0.02919 | 0.02713 | 2.086·10^-6^ | 6.611·10^-5^ | 0.006547 | 0.01087 |
| Sy.x | 0.08543 | 0.08236 | 0.001021 | 0.005749 | 0.04046 | 0.05214 |

**Table S12.** Output parameters for the fitting of T_1ρ_ signal decay for water-washed cell wall material from *S*. *commune* MM-N static cultures.

|  | **Ch2** | **Ch8** | **A1** | **A3** | **B3** | **B5** | **NRb3/5** |
| --- | --- | --- | --- | --- | --- | --- | --- |
| Preferred model | One phase decay | One phase decay | One phase decay | One phase decay | Two phase decay | One phase decay | One phase decay |
| p-value | 0.1314 | 0.0423 | 0.0628 | 0.1418 | 0.0082 | 0.7416 | 0.2944 |
| t½ (ms) | 8.071 | 13.94 | 13.12 | 13.75 | - | 2.36 | 1.739 |
| t½ error (ms) | 2.281 | 4.476 | 3.949 | 4.637 | - | 0.2064 | 0.08471 |
| PercentFast | - | - | - | - | 81.58 | - | - |
| PercentFast error | - | - | - | - | 2.865 | - | - |
| t½ slow (ms) | - | - | - | - | 22.7 | - | - |
| t½ slow error (ms) | - | - | - | - | 10.26 | - | - |
| t½ fast (ms) | - | - | - | - | 1.11 | - | - |
| t½ fast error (ms) | - | - | - | - | 0.06789 | - | - |
| R squared | 0.8333 | 0.7517 | 0.782 | 0.734 | 0.9996 | 0.9904 | 0.9971 |
| Sum of Squares | 0.0517 | 0.04836 | 0.04323 | 0.05591 | 0.0002387 | 0.007003 | 0.00209 |
| Sy.x | 0.1137 | 0.11 | 0.104 | 0.1182 | 0.01092 | 0.04184 | 0.02286 |

**Table S13.** Output parameters for the fitting of water-edited signal buildup for water-washed cell walls from *S*. *commune* MM-N liquid shaken cultures.

|  | **Ch2** | **Ch8** | **M6** | **A1** | **A3** |
| --- | --- | --- | --- | --- | --- |
| t½ (ms) | 1.686 | 1.726 | 1.137 | 1.648 | 1.826 |
| t½ error (ms) | 0.2424 | 0.2147 | 0.1009 | 0.2681 | 0.3074 |
| R-squared | 0.9194 | 0.9494 | 0.9502 | 0.9162 | 0.9226 |
| Sum of Squares | 0.0397 | 0.06422 | 0.04856 | 0.1318 | 0.125 |
| Sy.x | 0.07045 | 0.07316 | 0.06361 | 0.1048 | 0.1021 |

**Table S14.** Output parameters for the fitting of water-edited signal build-up for water-washed cell wall material from *S*. *commune* grown on MM-N under static conditions.

|  | **Ch2** | **Ch8** | **A1** | **A3** | **M6** | **B3** |
| --- | --- | --- | --- | --- | --- | --- |
| t½ (ms) | 1.591 | 1.926 | 1.723 | 1.879 | 1.204 | 1.76 |
| t½ error (ms) | 0.2169 | 0.267 | 0.2917 | 0.3409 | 0.09176 | 0.3084 |
| R-squared | 0.934 | 0.9464 | 0.9154 | 0.9156 | 0.9645 | 0.913 |
| Sum of Squares | 0.08784 | 0.07121 | 0.1327 | 0.1402 | 0.03391 | 0.1386 |
| Sy.x | 0.08556 | 0.07703 | 0.1052 | 0.1081 | 0.05315 | 0.1075 |

**References**

Chakraborty, A., Fernando, L.D., Fang, W., Dickwella Widanage, M.C., Wei, P., Jin, C., Fontaine, T., Latge, J.P., Wang, T., 2021. A molecular vision of fungal cell wall organization by functional genomics and solid-state NMR. Nat Commun 12, 6346.

Ehren, H.L., Appels, F.V.W., Houben, K., Renault, M.A.M., Wösten, H.A.B., Baldus, M., 2020. Characterization of the cell wall of a mushroom forming fungus at atomic resolution using solid-state NMR spectroscopy. Cell Surf 6, 100046.

Kleijburg, F.E.L., 2024. Functions of cell wall components of *Schizophyllum commune*, Universiteit Utrecht, Utrecht.

Kleijburg, F.E.L., Safeer, A.A., Baldus, M., Wösten, H.A.B., 2023. Binding of micro-nutrients to the cell wall of the fungus *Schizophyllum commune*. Cell Surf 10, 100108.

Lee, W., Tonelli, M., Markley, J.L., 2015. NMRFAM-SPARKY: enhanced software for biomolecular NMR spectroscopy. Bioinformatics 31, 1325-1327.

Safeer, A., Kleijburg, F., Bahri, S., Beriashvili, D., Veldhuizen, E.J.A., van Neer, J., Tegelaar, M., de Cock, H., Wösten, H.A.B., Baldus, M., 2023. Probing Cell-Surface Interactions in Fungal Cell Walls by High-Resolution 1H-Detected Solid-State NMR Spectroscopy. Chemistry 29, e202202616.
